# Light-driven flow synthesis of acetic acid from methane with chemical looping

**DOI:** 10.1038/s41467-023-38731-y

**Published:** 2023-05-26

**Authors:** Wenqing Zhang, Dawei Xi, Yihong Chen, Aobo Chen, Yawen Jiang, Hengjie Liu, Zeyu Zhou, Hui Zhang, Zhi Liu, Ran Long, Yujie Xiong

**Affiliations:** 1 Hefei National Research Center for Physical Sciences at the Microscale, Collaborative Innovative Center of Chemistry for Energy Materials (iChEM), Key Laboratory of Precision and Intelligent Chemistry, School of Chemistry and Materials Science, National Synchrotron Radiation Laboratory, School of Nuclear Science and Technology, University of Science and Technology of China, Hefei, Anhui 230026 China; 2 Institute of Energy, Hefei Comprehensive National Science Center, 350 Shushanhu Rd, Hefei, Anhui 230031 China; 3 School of Physical Science and Technology, ShanghaiTech University, Shanghai, 201203 China; 4 State Key Laboratory of Functional Materials for Informatics, Shanghai Institute of Microsystem and Information Technology, Chinese Academy of Sciences, Shanghai, 200050 China; 5 Anhui Engineering Research Center of Carbon Neutrality, College of Chemistry and Materials Science, Key Laboratory of Functional Molecular Solids, Ministry of Education, Anhui Normal University, Wuhu, Anhui 241002 China

**Keywords:** Catalytic mechanisms, Characterization and analytical techniques, Photocatalysis

## Abstract

Oxidative carbonylation of methane is an appealing approach to the synthesis of acetic acid but is limited by the demand for additional reagents. Here, we report a direct synthesis of CH_3_COOH solely from CH_4_ via photochemical conversion without additional reagents. This is made possible through the construction of the PdO/Pd–WO_3_ heterointerface nanocomposite containing active sites for CH_4_ activation and C–C coupling. In situ characterizations reveal that CH_4_ is dissociated into methyl groups on Pd sites while oxygen from PdO is the responsible for carbonyl formation. The cascade reaction between the methyl and carbonyl groups generates an acetyl precursor which is subsequently converted to CH_3_COOH. Remarkably, a production rate of 1.5 mmol g_Pd_^–1^ h^–1^ and selectivity of 91.6% toward CH_3_COOH is achieved in a photochemical flow reactor. This work provides insights into intermediate control via material design, and opens an avenue to conversion of CH_4_ to oxygenates.

## Abstract (web summary)

A light-driven, chemical looping approach to CH_3_COOH synthesis from methane using a Pd/PdO–WO_3_ nanocomposite is reported. Methane is converted to methyl and carbonyl intermediates at the Pd and PdO sites, respectively, to produce CH_3_COOH.

## Introduction

Methane conversion into value-added chemicals under mild condition is a promising strategy for maximizing CH_4_ utilization and mitigating the greenhouse effect^1–5^. In particular, the partial oxidation of CH_4_ at low temperature (<200 °C) is an attractive approach to generate valuable oxygenates (e.g., CH_3_OH, HCHO, HCOOH and CH_3_COOH) while reducing energy input and carbon emission in traditional gas-phase CH_4_ conversion^6–9^. Among the products, acetic acid (CH_3_COOH) is an important feedstock of chemical industries. Generally, the synthesis of CH_3_COOH from CH_4_ requires a three-step process involving the production of syngas and methanol, which suffers from extra resource consumption and safety issues^10^. It is thus imperative to develop a green synthetic approach that can directly convert CH_4_ to CH_3_COOH. Although recent reports have demonstrated the oxidative carbonylation of CH_4_ to CH_3_COOH in thermocatalytic processes, the requirement for additional oxidants (e.g., O_2_ and H_2_SO_4_) and/or CO limits their further applications^11,12^. Moreover, multiple side reactions take place in various reactants to generate undesired products such as HCOOH and CO_2_, and further limit the selectivity to CH_3_COOH^13^.

Intuitively, photocatalysis should be a potential approach to the green transformation of CH_4_, in which the ·OH radical derived from water oxidation is the ideal substitute for additional oxidant^14,15^. In fact, the metal-decorated semiconductor photocatalysts, which offer synergistic effects between metal and semiconductor on electronic structure, charge separation and intermediate adsorption, have been demonstrated to be effective for CH_4_ activation^16–18^. For instance, Pd-based photocatalysts have been reported for the conversion of CH_4_ into C_1_ oxygenate products (CH_3_OH, CH_3_OOH, HCHO, etc.)^19^. The ·OH radical produced from photocatalytic water oxidation enables the activation of CH_4_ to generate methyl intermediates (*CH_3_), which can be stabilized on Pd sites for further reactions^20,21^. However, it still remains a grand challenge to achieve the photocatalytic production of CH_3_COOH, mainly due to the difficulties of forming carbonyl intermediates and controlling methyl−carbonyl coupling in photocatalysis. The formation of carbonyl intermediates is the key to the production of CH_3_COOH using CH_4_ as the sole carbon source, which raises very high requirements for rational construction of catalytically active sites on photocatalysts. Once carbonyl intermediates can be formed from CH_4_, the carbonylation of CH_4_ to CH_3_COOH would no longer need the addition of CO reagent. As demonstrated in thermocatalysis^22^, the carbonyl group generated in situ from CH_4_ oxidation can be coupled with the adsorbed *CH_3_, leading to CH_3_COOH formation.

Here, we report that CH_4_ as the sole carbon source can be directly converted to CH_3_COOH without additional reagents, by rationally integrating the catalytically active sites for CH_4_ activation and C–C coupling on material surface. The key is the construction of Pd/PdO heterostructure on WO_3_ support. The photogenerated holes in WO_3_ enable oxidation of H_2_O to ·OH radicals for CH_4_ activation while Pd sites stabilize *CH_3_ for further conversion. More importantly, PdO—the active species for CH_4_ oxidation through the Mar−van Krevelen mechanism^23^—is regarded as the key component for the transformation of CH_4_ to carbonyl intermediate (*CO) under light irradiation. As such, the carbonylation of CH_4_ can be achieved through the coupling of methyl and carbonyl intermediates, forming acetyl (CH_3_CO*) precursor toward the final product of CH_3_COOH. To facilitate the continuous reaction between methyl and carbonyl intermediates, we design a photochemical flow reaction device with arc-shaped flow channel, in which the flux of *CH_3_ can react with *CO intermediate continually by fully utilizing PdO and *CH_3_, to perform the cascade reaction. As a result, the PdO/Pd–WO_3_ heterointerface nanocomposite with optimal PdO content enables the remarkable selectivity of 91.6% and production rate of 1.5 mmol g_Pd_^–1^ h^–1^ toward CH_3_COOH, providing a feasible strategy for scale-up CH_4_ conversion.

## Results

### Structural characterization of nanocomposites

In the preparation of nanocomposites, Pd nanoparticles (NPs) are loaded on WO_3_ nanosheets (Pd/WO_3_), followed by the further thermal annealing process to decorate PdO species on Pd NPs. The obtained samples are denoted as PdO/Pd–WO_3_-*x* where *x* = 1−5 by increasing the annealing temperature (refer to Methods). The Pd contents are kept constant in these samples, which are confirmed by inductively coupled plasma optical emission spectrometry (ICP-OES) (Supplementary Table 1), to exclude the effect of Pd content on CH_4_ conversion performance. Transmission electron microscopy (TEM) images reveal that the prepared WO_3_ nanosheets have the edge lengths of ~170 nm (Supplementary Fig. 1), and the nanoparticles in all samples are highly dispersed on WO_3_ substrate (Supplementary Fig. 2). The sizes of Pd NPs increase from Pd/WO_3_ to PdO/Pd–WO_3_-5 with the annealing temperature raised (Supplementary Fig. 3), implying the incorporation of oxygen atoms into the nanoparticles along with their lattice expansion. The samples are further characterized by X-ray diffraction (XRD) as shown in Supplementary Fig. 4. The diffraction peaks of Pd and PdO are absent in the XRD patterns, indicating that the nanoparticles are highly dispersed at a low loading amount.

**Fig. 1:**
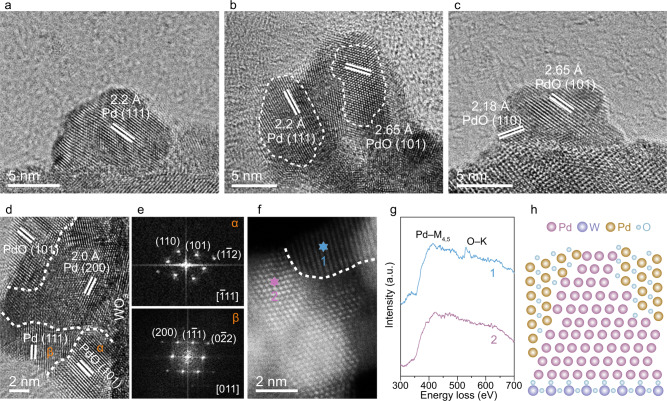
Structural characterization of nanocomposites. **a–c** HRTEM images of Pd/WO_3_ (**a**), PdO/Pd–WO_3_-2 (**b**) and PdO/Pd–WO_3_-5 (**c**). **d** Typical HRTEM image of PdO/Pd–WO_3_-2 sample showing Pd/PdO heterostructure. **e** Corresponding FFT patterns of α and β regions in **d**. **f** HAADF-STEM image of PdO/Pd–WO_3_-2 sample. **g** EELS spectra collected in the regions 1 and 2 marked in **f**. **h** Structural illustration of PdO–Pd–WO_3_ heterointerface.

To look into the detailed structures, the nanoparticles on WO_3_ supports are examined by high-resolution TEM (HRTEM). The Pd NPs are decorated with PdO with different oxidation degree by controlling the annealing temperature. As shown in Fig. 1a, the pristine Pd nanoparticle only displays the interplanar distance of 2.2 Å, corresponding to the spacing of Pd (111) planes^24–26^. After the annealing process, the new lattice fringes with a spacing of 2.65 Å appear in the nanoparticles (Fig. 1b and Supplementary Fig. 5), which can be assigned to the (101) planes of PdO^27,28^. Meanwhile, the Pd (111) planes are still observed in the nanoparticles, indicating the existence of Pd/PdO heterostructure in PdO/Pd–WO_3_-1 to PdO/Pd–WO_3_-4. As the annealing temperature reaches 450 °C, the Pd NPs are completely transformed to PdO NPs (Fig. 1c). Moreover, the compositions of Pd and PdO species are investigated by X-ray photoelectron spectroscopy (XPS). As shown in Supplementary Fig. 6, the content of Pd^2+^ increases by elevating the annealing temperature, in agreement with the findings from HRTEM images.

The Pd–PdO interface is further resolved meticulously to illustrate the active structure for CH_4_ conversion. Taking PdO/Pd–WO_3_-2 as an example, abundant Pd/PdO grain boundaries are observed by the distinguishable lattice parameters of Pd and PdO (Fig. 1d and Supplementary Fig. 7). Figure 1e shows the fast Fourier transform (FFT) diffraction patterns obtained from the α and β regions in Fig. 1d. The FFT pattern with the labels of (110), (101) and (1$\bar{1}$2) in α region fits the tetragonal structure of PdO along the zone axis of [$\bar{1}$11]^29^. Meanwhile, in β region, we can also obtain the FFT pattern of Pd along the zone axis of [011] direction belonging to the face-centered cubic (*fcc*) structure with (200), (1$\bar{1}$1) and (0$\bar{2}$2)^30^. Furthermore, the Pd/PdO grain boundary is examined by atomic-resolution high-angle annular dark-field scanning TEM (HAADF-STEM), with their compositions further analyzed via electron energy-loss spectroscopy (EELS). As shown in Fig. 1f and g, in addition to the detected signals of Pd–M_4,5_ edges at both sites 1 and 2, the peak of O–K edge is also recognized at site 1, corresponding to the composition of PdO^31,32^. Taken together, the aforementioned results demonstrate the existence of Pd/PdO heterostructure on WO_3_ support. During the annealing process, the PdO component appears on Pd NPs under the cooperation of oxygen and support, as nano-islands rather than core-shell structure^33^, forming the PdO–Pd–WO_3_ triple interface (Fig. 1h).

### Performance of light-driven CH_4_ conversion

**Fig. 2:**
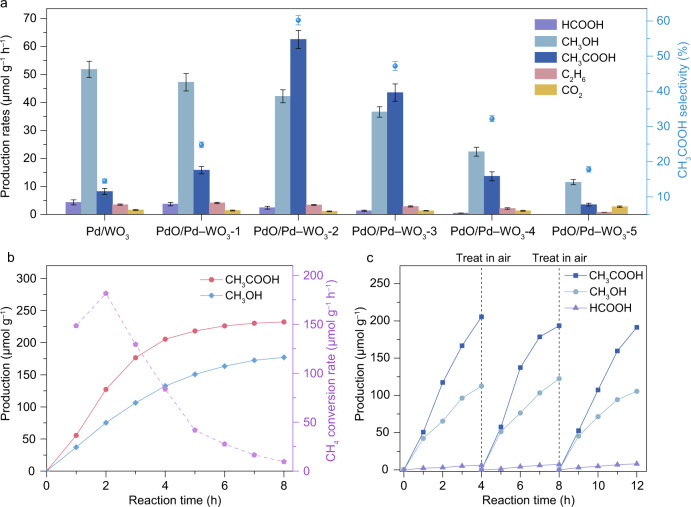
Light-driven CH_4_ conversion performance of nanocomposites. **a** Production rates for light-driven CH_4_ conversion over Pd/WO_3_ and PdO/Pd–WO_3_-1 to PdO/Pd–WO_3_-5 samples. **b** Time-dependent rates of CH_3_COOH and CH_3_OH production as well as CH_4_ conversion over PdO/Pd–WO_3_-2 nanocomposite. **c** Reaction-regeneration cycles in CH_4_ conversion. The error bars represent the standard deviation of the experiments.

Upon acquiring the fine structures, we are now in a position to investigate the efficacy of the PdO/Pd–WO_3_ nanocomposites in light-driven CH_4_ conversion. The photochemical measurements are conducted in a quartz reactor under xenon arc lamp irradiation. Pure WO_3_ nanosheets show sluggish properties for CH_4_ conversion (Supplementary Fig. 8). After Pd NPs deposition, the CH_4_ conversion activity over Pd/WO_3_ is enhanced with CH_3_OH as the primary product (Fig. 2a). Interestingly, the photochemical performance is significantly altered after the incorporation of PdO species into the Pd/WO_3_ structure, which exhibits a volcano-like relationship with the amount of PdO. Specifically, the addition of PdO to the samples dramatically boosts the production of CH_3_COOH. Among the samples, the PdO/Pd–WO_3_-2 achieves the highest production rate and selectivity toward CH_3_COOH at 62.5 μmol g^–1^ h^–1^ and 60.2%, respectively. The outstanding performance of converting CH_4_ to CH_3_COOH indicates that the Pd/PdO heterostructure enables an efficient C–C coupling process. However, the excessive PdO in nanocomposites hinders the Schottky contact between Pd and WO_3_, which further reduces photo-induced charge separation efficiency and substantially suppresses photochemical performance (Supplementary Figs. 9 and 10).

To confirm the carbon source of the liquid products, the origin of CH_3_OH and CH_3_COOH, as the main products, are traced with ^13^C nuclear magnetic resonance (^13^C NMR) spectroscopy by using ^13^CH_4_ as the reactant. As shown in Supplementary Fig. 11, the peaks at 20.5 and 176.7 ppm are attributed to ^13^CH_3_^13^COOH while the peak at 48.9 ppm is assigned to ^13^CH_3_OH. In addition, the control experiments indicate that no product can be detected in the absence of nanocomposite, light irradiation or CH_4_ reactant (Supplementary Fig. 12). These results provide the evidence that the primary products indeed originate from light-driven CH_4_ conversion.

It is worth pointing out that differing from the conventional CH_4_ photooxidation requiring extra oxidant addition (e.g., O_2_), our reaction system utilizes the reactants of CH_4_ and H_2_O, in which the ·OH radical produced from water oxidation is the ideal oxidizer for CH_4_ activation (Supplementary Figs. 13 and 14)^34^. As displayed in Supplementary Fig. 15, increasing the concentration of O_2_ will not promote the generation of liquid products but lead to CO_2_ production during the CH_4_ photooxidation over PdO/Pd–WO_3_-2. The activation of CH_4_ by ·OH radicals can produce ·CH_3_ as detected by in situ electron paramagnetic resonance (EPR) measurement (Supplementary Fig. 16).

To better evaluate the efficiency, we conduct time-dependent measurement over PdO/Pd–WO_3_-2. As shown in Fig. 2b, the production of CH_3_COOH and CH_3_OH gradually increases in the first 3 h, achieving an impressive CH_4_ conversion rate of 181.5 μmol g^–1^ h^–1^. However, the performance shows distinct decay when the reaction time exceeds 3 h, which should be ascribed to the consumption of PdO species after the photochemical process (Supplementary Figs. 17 and 18). The constructed Pd/PdO heterostructure is gradually destroyed along with the reaction, which further reduces the efficiency of C–C coupling toward CH_3_COOH production. In the meantime, negligible H_2_ detection during the reaction suggests that the lattice oxygen of WO_3_ is consumed for H_2_O production, which also leads to performance decay (Supplementary Figs. 19 and 20). To overcome this limitation, we carry out the regeneration process by heating the nanocomposites in air, in which the consumed oxygen (i.e., PdO on Pd and lattice oxygen in WO_3_) can be replenished to recover activity (Fig. 2c and Supplementary Fig. 21). As such, a durable photochemical CH_4_ conversion process can be established by recycling the photochemical CH_4_ conversion and air recovery. The durability measurement indicates that the performance of PdO/Pd–WO_3_-2 is well maintained for five cyclic tests with each cycle lasting 5 h in such a recycling system (Supplementary Fig. 22). Moreover, the leaching out of Pd during the cyclic tests is also negligible according to the results of mass spectrometry (Supplementary Fig. 23).

### Reaction intermediates detection

**Fig. 3:**
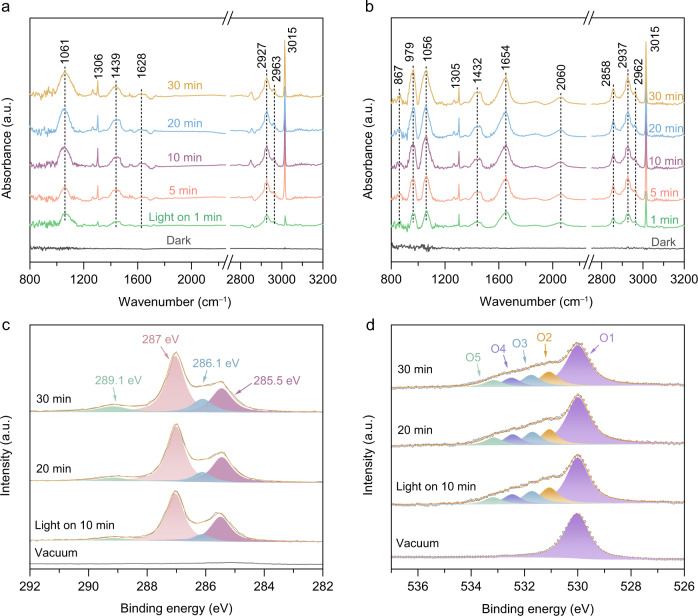
In situ characterizations of the photochemical CH_4_ conversion process. **a**, **b** In situ DRIFT spectra for light-driven CH_4_ conversion over Pd/WO_3_ (**a**) and PdO/Pd–WO_3_-2 (**b**). **c**, **d** In situ NAP-XPS results of high-resolution C 1 *s* (**c**) and O 1 *s* (**d**) spectra over PdO/Pd–WO_3_-2 nanocomposite.

The information gleaned above has recognized the promising performance for the conversion of CH_4_ to CH_3_COOH by modulating Pd/PdO heterostructure. Naturally, a question arises how CH_4_ evolves into CH_3_COOH over PdO/Pd–WO_3_ nanocomposites without additional carbon sources. To this end, we investigate the reaction intermediates over the nanocomposites during the photochemical CH_4_ conversion process. Figure 3a shows the in situ diffuse reflectance-infrared Fourier-transform spectra (DRIFTS) for light-driven CH_4_ conversion over Pd/WO_3_ sample. Upon light irradiation, apart from the peaks at 1305 and 3015 cm^–1^ corresponding to C–H deformation vibration of CH_4_, the peak at 1439 cm^–1^ for CH_2_/CH_3_ deformation vibration appears gradually, indicating the CH_4_ dissociation over the sample^35,36^. Moreover, the significant growth of vibrational peak at 1061 cm^–1^ and bands at 2927 and 2963 cm^–1^, corresponding to the methoxy and C–H stretching vibrations in CH_3_OH product, can be attributed to the CH_4_ activation in the presence of ·OH^37^. In sharp contrast, PdO/Pd–WO_3_ nanocomposites that can produce CH_3_COOH through light-driven CH_4_ conversion exhibit different behavior in DRIFTS (Fig. 3b). In addition to the vibration signals of CH_3_OH observed over Pd/WO_3_, the additional vibrational modes of C = O (1654 cm^–1^), C–O (979 cm^–1^), C–C (867 cm^–1^) and C–H (2858 cm^–1^) stretching vibrations can be monitored for the formation of CH_3_COOH over PdO/Pd–WO_3_-2^38,39^. Notably, a broad peak at 2060 cm^–1^ in Fig. 3b is observed with the light irradiation proceeding, which can be assigned to the adsorbed *CO on Pd site (Supplementary Fig. 24)^40^. The vibration signals of C = O and *CO only appear with the existence of PdO species, implying that the synergistic effect of Pd/PdO heterostructure in the nanocomposite can facilitate the CH_3_COOH production with *CO as an intermediate.

To further understand the process with elemental information, the surface carbon and oxygen species are also monitored by in situ near ambient pressure XPS (NAP-XPS) characterization. As shown in Fig. 3c, after introducing the reactant into NPA-XPS chamber, the peak of gas-phase CH_4_ (287.0 eV) is observed in the high-resolution C 1 *s* XPS spectrum (Supplementary Fig. 25). Upon light irradiation, three C 1 *s* peaks of surface ·CH_*x*_ (285.5 eV), C–O (286.1 eV) and COO (289.1 eV) species appear and increase with the time evolution^41–43^. Meanwhile, the formation of oxygenates from CH_4_ oxidation is also verified by collecting the O 1 *s* spectra in NAP-XPS studies. Apart from the peak of lattice oxygen in sample (**O1**, 530 eV), the featured peaks of hydroxyl (**O2**, 531.1 eV), C–O (**O3**, 531.8 eV), adsorbed H_2_O (**O4**, 532.5 eV) and C = O (**O5**, 533.2 eV) species are resolved after light illumination (Fig. 3d)^44–46^. Of note, although the signals of *CO have been detected by in situ DRIFT and NAP-XPS measurements, gaseous CO is not observed as a product. Indeed, previous work has demonstrated that the adsorption of CO on PdO site is extremely strong so that *CO would be coupled with other intermediates before desorption^47^. Apparently, the surface ·CH_*x*_, C–O and C = O species are corroborated with the observation of in situ DRIFTS spectra. This indicates that the co-adsorption of CH_4_ and H_2_O over Pd/PdO heterostructure can produce various surface carbonaceous intermediates including methyl and carbonyl species and further generate liquid oxygenates.

### Mechanistic study

**Fig. 4:**
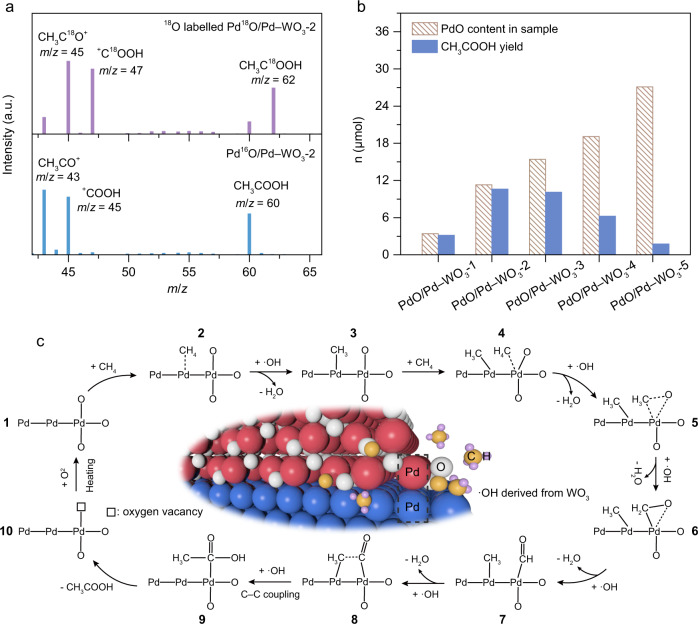
Mechanism for photochemical CH_4_ to CH_3_COOH conversion. **a** Mass spectra of CH_3_COOH product using Pd^16^O/Pd–WO_3_-2 and ^18^O-labeled Pd^18^O/Pd–WO_3_-2 nanocomposite. **b** The comparison of PdO contents in samples (50 mg) and the CH_3_COOH yields of the corresponding samples. **c** Schematic illustration for photochemical conversion of CH_4_ to CH_3_COOH over Pd/PdO heterointerface in the presence of ·OH radicals. The numbers represent the reaction steps.

As revealed by in situ characterizations, the carbonyl species is the key intermediate for the conversion of CH_4_ to CH_3_COOH, which is closely correlated with the presence of PdO in the prepared nanocomposite. The case of Pd/WO_3_ reveals that the generation of ·OH radicals alone cannot lead to the formation of C = O in the absence of PdO species (Fig. 3a), implying that the oxygen in C = O is most likely derived from PdO in the nanocomposite. To further trace the oxygen source of carbonyl intermediate in CH_3_COOH production, we prepare the ^18^O-labeled PdO-modified nanocomposite (denoted as Pd^18^O/Pd–WO_3_-2) by annealing pristine Pd/WO_3_ in ^18^O_2_ atmosphere. Subsequently, the light-driven CH_4_ oxidation is performed over Pd^18^O/Pd–WO_3_-2 and the products are analyzed by gas chromatography−mass spectrometry (GC−MS), in reference to Pd^16^O/Pd–WO_3_-2. In contrast to the case of Pd^16^O/Pd–WO_3_-2, the peaks at *m*/*z* = 45 and 47 by Pd^18^O/Pd–WO_3_-2 can be ascribed to CH_3_C^18^O^+^ and ^+^C^18^OOH (Fig. 4a), indicating that the O atom in *CO is derived from the lattice oxygen of Pd^18^O in sample. As a result, the peak at *m*/*z* = 62 for CH_3_C^18^OOH can be detected.

To further understand the working mechanism of PdO in the production of CH_3_COOH, we quantitatively establish the relation between PdO consumption and CH_3_COOH production. With this purpose, the H_2_-temperature-programmed reduction (H_2_-TPR) is employed to determine the content of PdO in PdO/Pd–WO_3_-*x* nanocomposites (Supplementary Fig. 26). As revealed in Fig. 4b, the amounts of PdO in samples are very close to the CH_3_COOH yield at low PdO contents (i.e., PdO/Pd–WO_3_-1 and PdO/Pd–WO_3_-2). Given that the PdO is completely consumed after photochemical tests, this further confirms that the lattice oxygen of PdO solely contributes to the formation of C = O in CH_3_COOH during photochemical CH_4_ oxidation process. However, excessive PdO in nanocomposites will lower the content of metallic Pd to form PdO−WO_3_ interface, which suppresses charge separation and reduces performance^48–50^. In this case, the low CH_4_ photooxidation performance allows most lattice oxygen atoms in PdO to remain in the samples (i.e., PdO/Pd–WO_3_-3 to PdO/Pd–WO_3_-5 in Fig. 4b). It is worth pointing out that the formation of Pd–PdO interface in nanocomposite is critical for CH_3_COOH generation. Our control experiments indicate that CH_3_COOH cannot be produced through simply mixing Pd/WO_3_ with PdO, suggesting that Pd–PdO interface is a key factor for CH_3_COOH generation (Supplementary Fig. 27). Moreover, the CH_3_COOH production depends on the structural character of Pd/PdO heterointerface (Supplementary Figs. 28 and 29), corroborating the importance of Pd/PdO interface quality to CH_3_COOH synthesis.

Taken together, the experimental results have revealed the critical role of Pd/PdO heterointerface in CH_4_-to-CH_3_COOH conversion. Figure 4c illustrates the proposed reaction pathway. CH_4_ prefers to be activated at Pd site in the presence of ·OH and form Pd–CH_3_ intermediate (Supplementary Fig. 30). The methyl species can be gradually converted to Pd−CO intermediate through the combination with O atom from PdO and the dehydrogenation by ·OH. Subsequently, the C–C coupling between carbonyl and methyl species generates the Pd–COCH_3_ intermediate at Pd−PdO interface, and the further hydrolysis of Pd–COCH_3_ gives CH_3_COOH as a product^51–53^. Of note, W sites are also active for *CH_3_ generation by directly oxidizing CH_4_ on WO_3_. However, the *CH_3_ formed on WO_3_ can hardly approach the *CO on PdO so that the surplus *CH_3_ species would evolve into C_1_ oxygenates^54,55^.

From the working mechanism of Pd/PdO heterostructure, we can now understand that the complete consumption of lattice oxygen in PdO during light-driven CH_3_COOH production, in the case of PdO/Pd–WO_3_-2, will inevitably lead to the significant performance decay for CH_3_COOH production after the reaction (Supplementary Fig. 31). In comparison, the CH_3_OH production is not significantly affected by the oxygen loss in PdO. It is worth noting that the evolution of WO_3_ in photochemical CH_4_ conversion is also a factor for performance decay. Given that no H_2_ is detected in the photochemical process, the WO_3_ is inevitably reduced by photo-induced electrons, which is accompanied with gradual lattice oxygen loss, also causing performance decay (Supplementary Fig. 20). Nevertheless, the amount of lost oxygen atoms in WO_3_ is determined to be 1.28% through the calculation based on the demand of ·OH radical production, which is negligible as compared with the consumption of PdO (taking PdO/Pd–WO_3_-2 as example). Furthermore, the lost lattice oxygen in WO_3_ can be replenished together with that in PdO during the regeneration process, recovering photochemical activity.

Following the mechanistic studies, our investigation on photochemical CH_4_ conversion in gas-solid phase indicates that the generated *CH_3_ may undergo self-coupling to produce C_2_H_6_ as the primary product (Supplementary Fig. 32). For this reason, the controllable utilization of *CH_3_ in solution and gas phases is of great importance to further improve the production rate and selectivity for CH_3_COOH.

### Photochemical flow synthesis of CH_3_COOH

The key to controllable *CH_3_ utilization is the efficient methyl−carbonyl coupling. Certainly, such an efficient coupling should be based on the supply of sufficient *CO species. Our control experiments show that the addition of CO to the reaction system using Pd/WO_3_ nanocomposite, in the absence of PdO, can deliver similar CH_3_COOH production (Supplementary Fig. 33). In comparison, the addition of methanol does not obviously promote the CH_3_COOH production over PdO/Pd–WO_3_-2 nanocomposite, suggesting that CH_3_COOH is not the product primarily from methanol carbonylation (Supplementary Fig. 34). The results provide us the clues for enhancing CH_3_COOH production—the cascade reaction between *CO and *CH_3_ on nanocomposite in continuous reaction channels that can promote the utilization of PdO and *CH_3_.

**Fig. 5:**
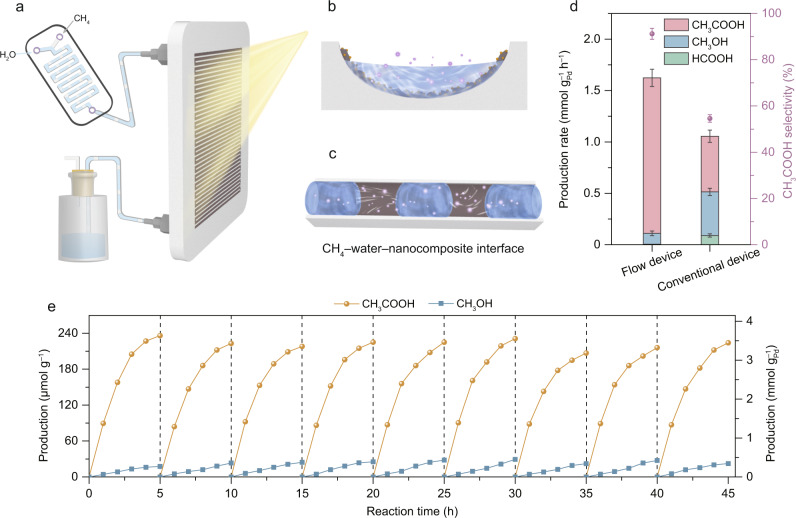
Photochemical flow synthesis of CH_3_COOH from CH_4_. **a** Schematic illustration of photochemical flow reaction device, including reactants supplier, homemade reactor and products collector. **b**, **c** Side (**b**) and top (**c**) views of the arc-shaped flow channel in homemade reactor and the three-phase contact between CH_4_, H_2_O and sample. The purple, blue and brown colors represent CH_4_, H_2_O and nanocomposite, respectively. **d** Production rate and selectivity of light-driven CH_4_ conversion toward CH_3_COOH over PdO/Pd–WO_3_-2 nanocomposite using the flow reaction device or conventional device for the first 3 h. **e** Reaction-regeneration cycles on PdO/Pd–WO_3_-2 sample by employing the flow reaction device. The error bars represent the standard deviation of the experiments.

To this end, we design a photochemical flow reaction device with arc-shaped flow channels to further enhance the performance of CH_3_COOH production (Supplementary Fig. 35). In this design, the *CH_3_ species that have not coupled with *CO can migrate along the sample to further react with the adsorbed *CO or even evolve into *CO on the downstream PdO sites, promoting the conversion of CH_4_ to CH_3_COOH. Specifically, CH_4_ and H_2_O are premixed to form the monodispersed gas bubbles, which are then pumped into the flow reactor to generate gas-liquid-solid contact in channels (Fig. 5a). Benefitting from the flowing reactants and three-phase interface between CH_4_, H_2_O and sample (Fig. 5b, c), the generated *CH_3_ in solution from gas-solid phase CH_4_ oxidation can be rapidly captured by *CO on sample layer to realize continuous synthesis of CH_3_COOH. As such, the remarkable selectivity of 91.6% and production rate of 90.7 μmol g^–1^ h^–1^ are achieved for CH_3_COOH production over PdO/Pd–WO_3_-2 nanocomposite (Fig. 5d). As normalized to the Pd loading weight, the production rate reaches 1.5 mmol g_Pd_^–1^, which exceeds the performance of existing photocatalysts for oxygenates production under mild condition (Supplementary Table 2). Furthermore, the integration of photochemical CH_4_ conversion with regeneration process also demonstrates the reproducibility and durability of the flow reaction device (Fig. 5e).

## Discussion

We have demonstrated a direct light-driven synthesis of CH_3_COOH solely from CH_4_ on PdO/Pd–WO_3_ heterointerface nanocomposite, by controlling carbonyl intermediate formation and methyl−carbonyl coupling. As revealed by solid evidence from in situ characterizations, the PdO species can convert CH_4_ into carbonyl intermediate, holding the key to CH_3_COOH production. Our isotope labeling experiments indicate that the oxygen atom in carbonyl intermediate is derived from the lattice oxygen of PdO in nanocomposite, providing important information for establishing a conversion−regeneration process toward long-term recyclability. Leveraging our understanding on CH_4_-to-CH_3_COOH conversion pathway, we have designed a photochemical flow reaction device enabling cascade reactions to enhance the efficiency and selectivity of acetic acid production. As a result, the approach achieves the impressive production rate of 1.5 mmol g_Pd_^–1^ h^–1^ and selectivity of 91.6% toward CH_3_COOH. This work highlights the importance of rational heterostructure engineering to controlling intermediates evolution, and provides new insights for selective C_2+_ oxygenates synthesis using methane as resource under mild conditions.

## Methods

### Chemicals

Sodium tungstate dehydrate (Na_2_WO_4_·2H_2_O, 99.5%), citric acid (CA, 99.5%) and palladium chloride (PdCl_2_, 98% metals basis) were purchased from Aladdin. Sodium borohydride (NaBH_4_, 98%) was obtained from Sigma-Aldrich. Hydrochloric acid (HCl, 36 ~ 38%) and ascorbic acid (AA, 99.7%) were purchased from Sinopharm Chemical Reagent Co., Ltd. The water used in all experiments was deionized. All of the chemical reagents were used as received without further purification.

### Materials preparation

WO_3_ nanosheets were prepared by a two-step process. Typically, 1 mmol Na_2_WO_4_·2H_2_O and 1.5 mmol citric acid were dissolved in 30 mL H_2_O to form a transparent solution. Then 3 mL of HCl solution (6 M) was added into the solution with vigorous stirring for 30 min. The mixture was transferred into a 50 mL Teflon-lined autoclave and heated at 120 °C for 24 h. The resulting precursor of WO_3_·H_2_O nanosheets was centrifuged and washed with water several times, and dried in a vacuum oven. The WO_3_ nanosheets were obtained by calcinating the collected solid in air at 400 °C for 2 h. For the synthesis of Pd/WO_3_ nanocomposites, 60 mg WO_3_ nanosheets were dispersed in 30 mL water to form a homogeneous suspension. Then 6.5 mg of PdCl_2_ was dissolved in 0.5 mL HCl solution (10 mM), which was added into the WO_3_ suspension and further reacted with 5 mg of NaBH_4_. The slurry was washed with water three times and dried in a vacuum oven, producing Pd/WO_3_ sample. The PdO/Pd–WO_3_-1, PdO/Pd–WO_3_-2 and PdO/Pd–WO_3_-3 nanocomposites were obtained by calcinating the Pd/WO_3_ sample at 120 °C, 200 °C and 260 °C for 5 h, respectively, with a heating rate of 1 °C min^–1^ in air. The PdO/Pd–WO_3_-4 and PdO/Pd–WO_3_-5 nanocomposites were obtained by calcinating the Pd/WO_3_ sample at 350 °C and 450 °C for 3 h and 2 h, respectively, with a heating rate of 2 °C min^–1^ in air.

### Characterization

Powder XRD patterns were measured by Philips X’Pert Pro Super X-ray diffractometer with Cu-Kα radiation (*λ* = 1.54178 Å). XPS characterizations of the prepared samples were carried out on JPS-9010MC (JEOL, Japan) with a hemispherical electron energy analyzer (1486 eV Al Kα radiation). TEM images were taken on a Hitachi Model H-7700 microscope at 100 kV. HRTEM images were taken on a JEOL JEM-2100 field-emission higher-resolution transmission electron microscope at 200 kV. The aberration-corrected HAADF-STEM images and EELS analysis were collected on the JEOL ARM-200F field-emission transmission electron microscope operated at 200 kV. EPR spectra for radical detection were obtained on the JEOL JES-FA200 spectrometer.

### Photochemical CH_4_ conversion measurement

In a typical test, 10.0 mg of sample was dispersed in 10 mL water and added into a 30 mL custom-made quartz tube reactor. The light-driven CH_4_ conversion experiments were carried out in pure CH_4_ atmosphere (0.1 MPa) at room temperature. The reactor was irradiated by a 300 W xenon lamp (PLS-SXE300, Perfect light) with light intensity of 200 mW cm^–1^. The gas products were quantified by a gas chromatograph (GC, 7890B, Ar carrier, Agilent) equipped with thermal conductivity detector (TCD) and flame ionization detector (FID). Another GC (Techcomp GC-7900, China) equipped with a TDX-01 packed column was employed to measure the amounts of CO and CO_2_. The liquid products were quantified by ^1^H NMR (Bruker Avance, 600 MHz) with a water suppression pulse sequence. A certain concentration of dimethyl sulfoxide (DMSO) solution was used as external standard to calibrate the liquid products. The trapping experiments were performed by adding 1 mM K_2_Cr_2_O_7_, Na_2_C_2_O_4_ and salicylic acid into the reaction solution as photo-induced electron, hole and ·OH scavengers, respectively.

For using the designed photochemical flow device, 100 mg of sample was loaded on the channel of the homemade flow reactor. The reactor was clamped with mould and quartz plate. The reactants of CH_4_ and H_2_O were premixed by the microfluidic device to form the monodisperse gas and bubble, which were then pumped into the reactor for photochemical conversion under 300 mW cm^–1^ of light irradiation. The liquid products were received in bottle. For recovering the photochemical performance, the sample was calcinated at 230 °C for 3 h with a heating rate of 1 °C min^–1^ in air.

### Isotope-labeling experiments

The isotope-labeling experiments were performed by using pure ^13^CH_4_ and ^12^CH_4_ as feeding gas. The liquid products were detected by ^13^C NMR. To trace the oxygen atom of CH_3_COOH, the PdO species in nanocomposite was generated by calcinating Pd/WO_3_ nanocomposite in ^18^O_2_ atmosphere at 200 °C for 8 h to label the oxygen atoms in PdO. The photochemical tests were performed in the homemade flow reaction device for maximizing CH_3_COOH yield. The CH_3_COOH product was concentrated and then analyzed by GC−MS (7890 A and 5975 C, He carrier, Agilent).

### Photocurrent measurements

The photocurrent tests of the prepared samples were conducted on CHI 660D electrochemical workstation (CH Instruments) with three-electrode system under light or dark condition. Typically, 5.0 mg of material was dispersed in 500 μL of ethanol/water mixture (4:1, v/v) and then dropped onto a 1 × 3 cm fluorine-doped tin oxide (FTO)-coated glass for work electrode preparation. The Pt foil and saturated Ag/AgCl electrode were employed as counter and reference electrode, respectively. The measurements were performed using 0.5 M Na_2_SO_4_ aqueous solution as electrolyte. The photocurrent responses of the photoelectrodes (i.e., I–t curves) were collected by measuring the photocurrent densities under chopped light irradiation (light on/off cycles: 10 s) at a bias potential of 0.8 V vs. Ag/AgCl.

### Detection of hydroxyl and methyl radicals

Briefly, the sample and 5,5-dimethyl-1-pyrroline N-oxide (DMPO) were dispersed in ice-bath water. The mixture was vigorously shaken and irradiated by using a 500 W xenon lamp, and then analyzed by EPR spectroscopy. Methyl radical was trapped by the same procedure under pure CH_4_ in the reaction system.

### In situ DRIFTS for photochemical CH_4_ conversion

In situ DRIFTS measurements were performed at BL01B in the NSRL in Hefei, China. The spectra were collected by using a Bruker IFS 66 v Fourier-transform spectrometer equipped with Harrick diffuse reflectance accessory with ZnSe and quartz window. Each spectrum was recorded by averaging 128 scans at a resolution of 2 cm^–1^. After sample loading, pure CH_4_ (99.999%) and water vapor were introduced into the chamber for background spectra collection. After that, the system was exposed to light irradiation and the spectra were collected when the irradiation times were 1, 5, 10, 20 and 30 min, respectively.

### In situ NAP-XPS measurement for photochemical CH_4_ conversion

In situ NAP-XPS measurements were carried at the beamline BL02B1 of SSRF under light irradiation or dark condition. The sample was dropped onto a silicon wafer and subsequently cleaned by Ar plasmon for 10 min to remove the surface agent on sample. The prepared sample was stored in the vacuum before the measurement. The XPS spectra were recorded under dark condition firstly. After that, the reactant was sequentially introduced into the analysis chamber with the partial pressure up to 45 Pa. Subsequently, the in situ NAP-XPS spectra were collected under 365 nm LED light irradiation.

## Supplementary information

Supplementary Information

Peer Review File

## Data Availability

The authors declare that all data supporting the findings of this study are available in the article and its Supplementary Information. Source data are provided with this paper. Figure 1g, Fig. 2a−c, Fig. 3a−d, Fig. 4a−b, Fig. 5d−e, Fig. S4, Fig. S6, Fig. S8, Fig. S9, Fig. S11, Fig. S17, Fig. S19, Fig. S22, Fig. S24. Additional data are available from the corresponding author upon reasonable request. Source data are provided with this paper.

## Source data

Source Data
