# Supplementary material for: Light-driven flow synthesis of acetic acid from methane with chemical looping
Source: Nat Commun. 2023 May 26;14:3047. doi: 10.1038/s41467-023-38731-y (PMC10220010; doi:10.1038/s41467-023-38731-y)
Supplement: Supplementary file 1 — Supplementary Information [file 41467_2023_38731_MOESM1_ESM.pdf]

Supplementary Information

Light-driven flow synthesis of acetic acid from methane with chemical looping

Wenqing Zhang[1,2], Dawei Xi[1], Yihong Chen[1], Aobo Chen[1], Yawen Jiang[1], Hengjie Liu[1], Zeyu Zhou[3,4], Hui Zhang[3,4], Zhi Liu[3,4], Ran Long[1]* and Yujie Xiong[1,2,5]*

[1]Hefei National Research Center for Physical Sciences at the Microscale, Collaborative Innovative Center of Chemistry for Energy Materials (iChEM), Key Laboratory of Precision and Intelligent Chemistry, National Synchrotron Radiation Laboratory, School of Chemistry and Materials Science, National Synchrotron Radiation Laboratory, School of Nuclear Science and Technology, University of Science and Technology of China, Hefei, Anhui 230026, China.

[2]Institute of Energy, Hefei Comprehensive National Science Center, 350 Shushanhu Rd. Hefei, Anhui 230031, China

[3]School of Physical Science and Technology, ShanghaiTech University, Shanghai 201203, China.

[4]State Key Laboratory of Functional Materials for Informatics, Shanghai Institute of Microsystem and Information Technology, Chinese Academy of Sciences, Shanghai 200050, China.

[5]Anhui Engineering Research Center of Carbon Neutrality, College of Chemistry and Materials Science, Key Laboratory of Functional Molecular Solids, Ministry of Education, Anhui Normal University, Wuhu, Anhui 241002, China.

*Corresponding author.
Tel: 0086-0551-63606657, E-mail: longran@mail.ustc.edu.cn; yjxiong@ustc.edu.cn

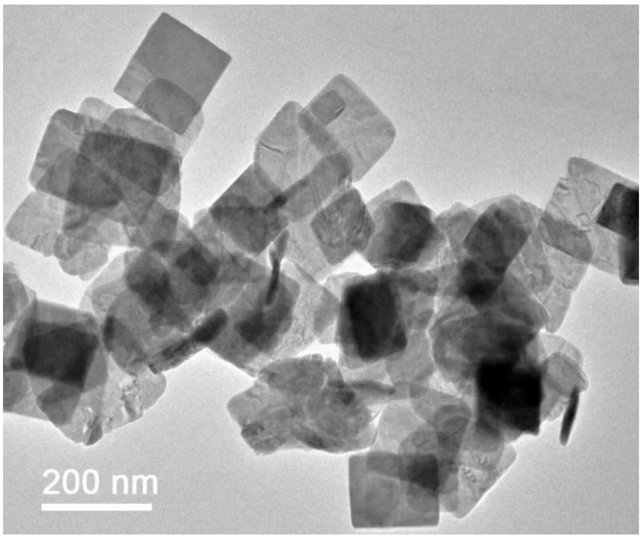

Supplementary Fig. 1 TEM image of WO_3 nanosheets.

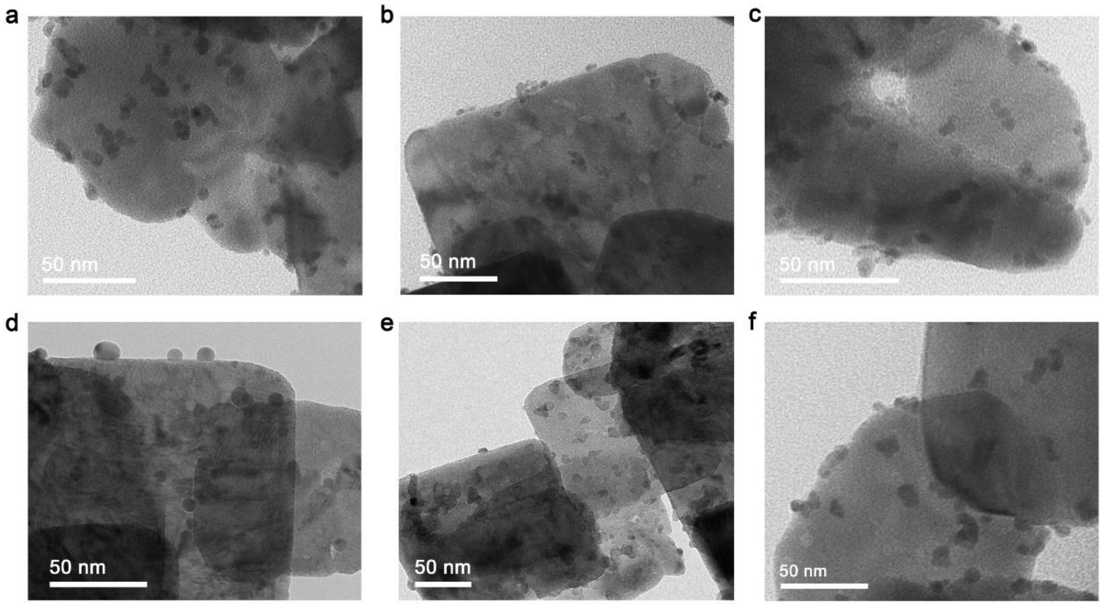

Supplementary Fig. 2 TEM images of (a) Pd/WO_3, (b) PdO/Pd–WO_3-1, (c) PdO/Pd–WO_3-2, (d) PdO/Pd–WO_3-3, (e) PdO/Pd–WO_3-4 and (f) PdO/Pd–WO_3-5 samples.

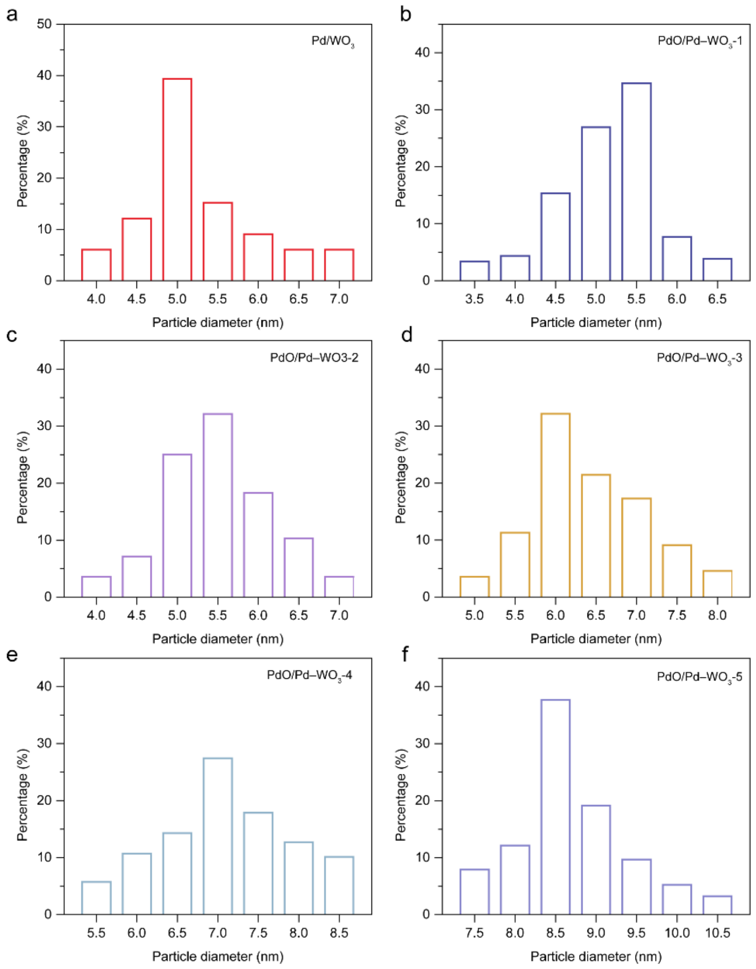

Supplementary Fig. 3 Size distribution of Pd or Pd/PdO nanoparticles in (a) Pd/WO_3, (b) PdO/Pd–WO_3-1, (c) PdO/Pd–WO_3-2, (d) PdO/Pd–WO_3-3, (e) PdO/Pd–WO_3-4 and (f) PdO/Pd–WO_3-5 samples.

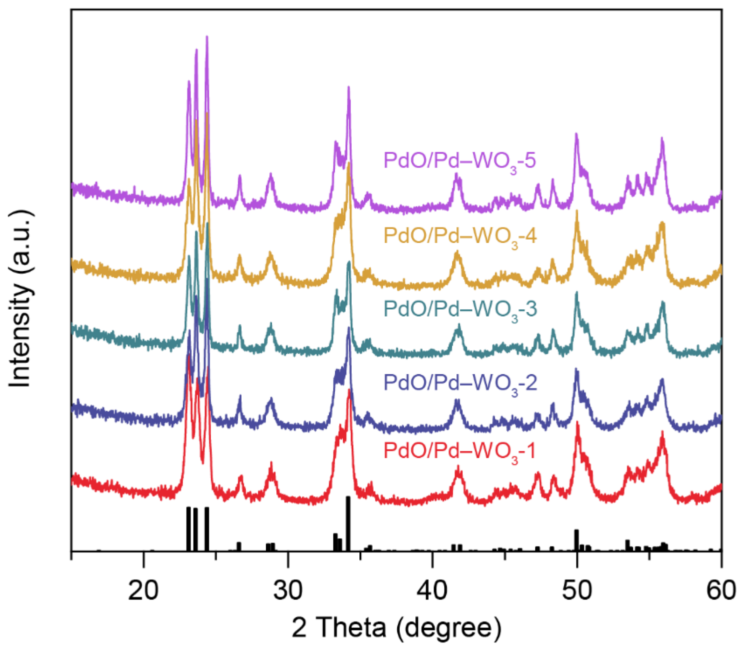

Supplementary Fig. 4 XRD patterns of PdO/Pd–WO_3-1 to PdO/Pd–WO_3-5 samples.

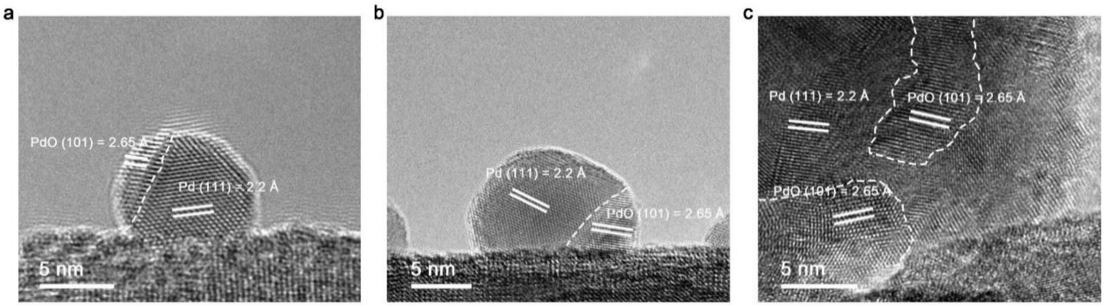

Supplementary Fig. 5 HRTEM images of (a) PdO/Pd–WO_3-1, (b) PdO/Pd–WO_3-3 and (c) PdO/Pd–WO_3-4 samples.

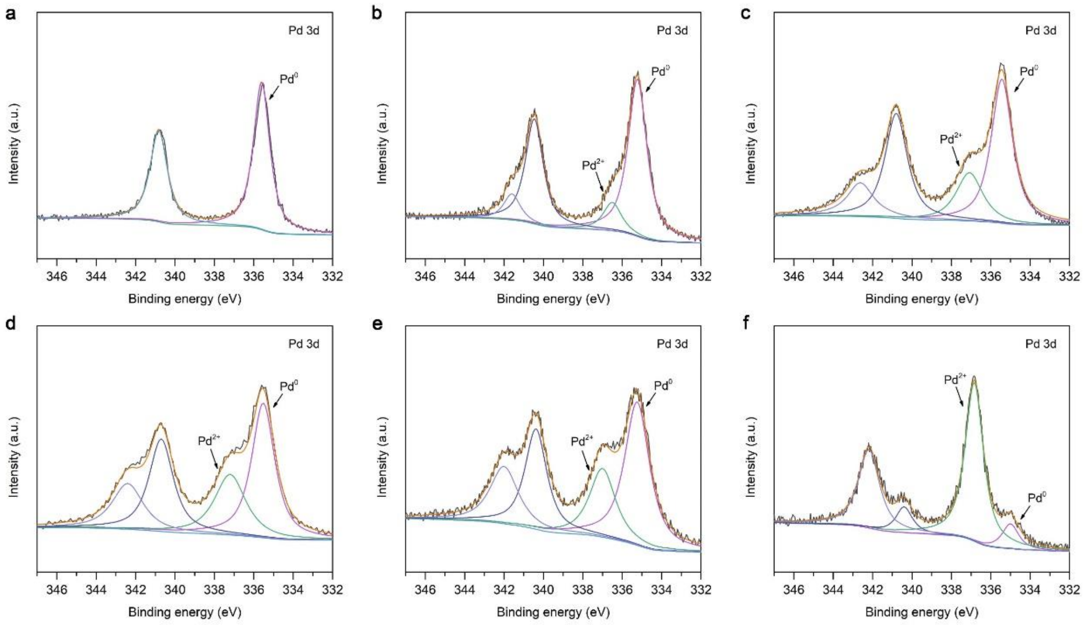

Supplementary Fig. 6 High-resolution Pd 3*d* XPS spectra of (a) Pd/WO_3, (b) PdO/Pd–WO_3-1, (c) PdO/Pd–WO_3-2, (d) PdO/Pd–WO_3-3, (e) PdO/Pd–WO_3-4 and (f) PdO/Pd–WO_3-5 samples.

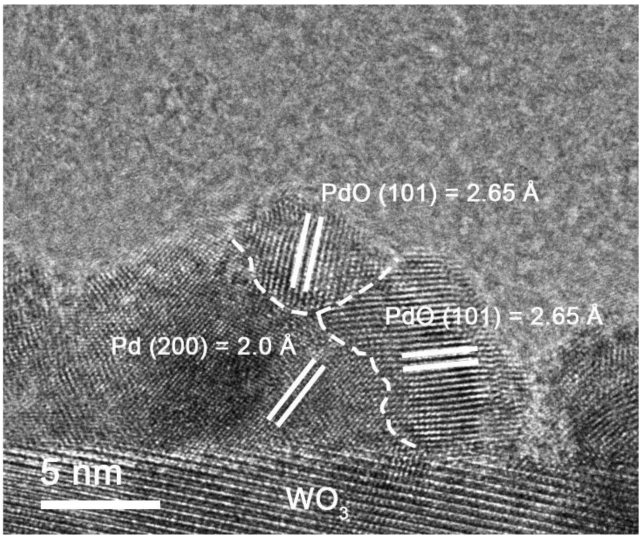

Supplementary Fig. 7 HRTEM images of PdO–Pd–WO_3 triple-phase interface.

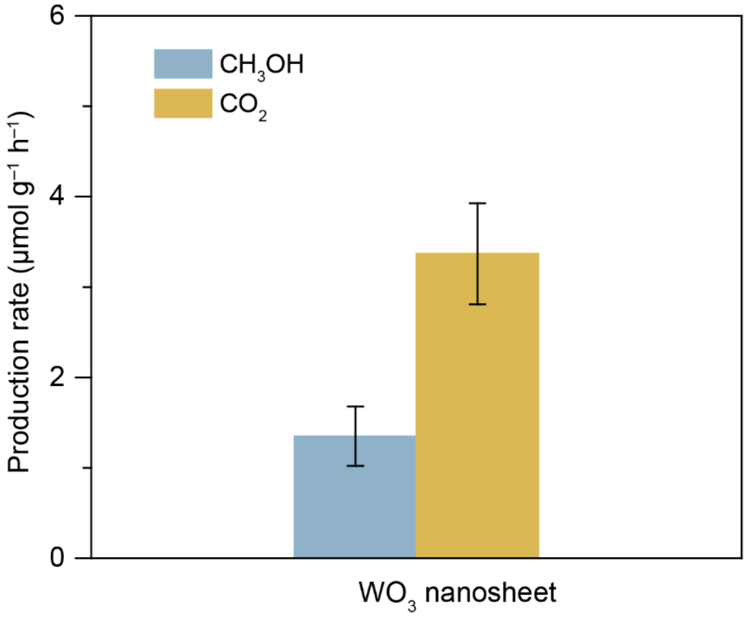

Supplementary Fig. 8 Production rates for photochemical CH_4 conversion over WO_3 nanosheets.

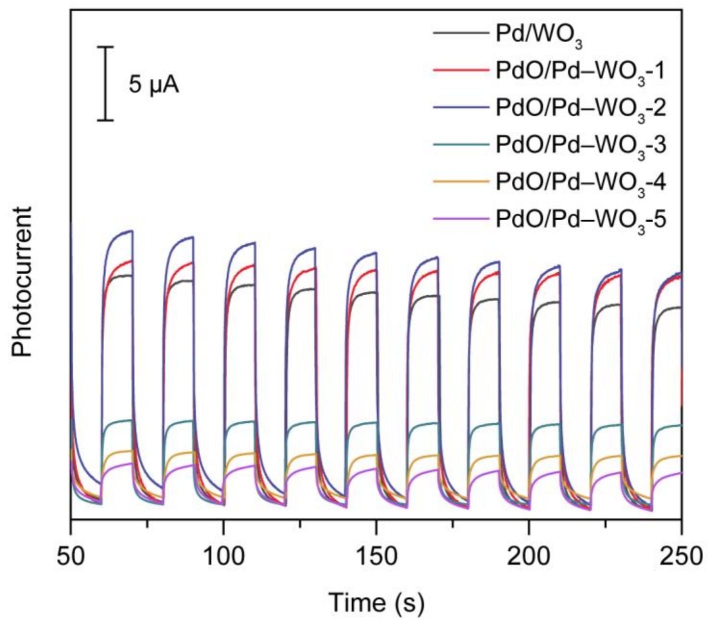

Supplementary Fig. 9 Photocurrent responses of the as-prepared samples.

As shown in Supplementary Fig. 9, the excessive PdO content in samples (PdO/Pd–WO_3-3, 4 and 5 samples) is detrimental to charge separation according to the reduced photocurrents.

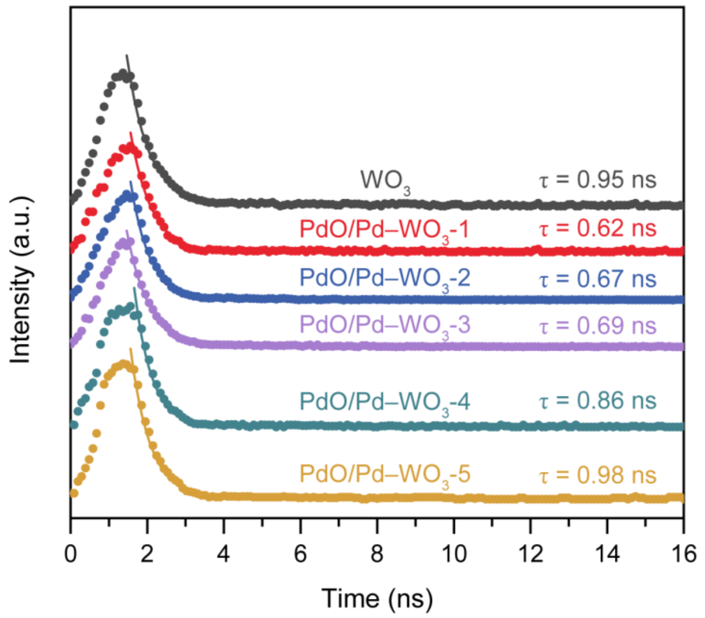

Supplementary Fig. 10 Time-resolved photoluminescence (TRPL) decay of the prepared samples.

As revealed in Supplementary Fig. 10, the average PL lifetimes are shortened from 0.95 ns for WO_3 to 0.62 ns for PdO/Pd–WO_3-1, which is attributed to the electron transfer channel from WO_3 to metallic Pd. Incorporating PdO into nanocomposites with appropriate content maintains the PL lifetimes (PdO/Pd–WO_3-2 and PdO/Pd–WO_3-3 samples). Nevertheless, the excessive PdO content shuts the channel and thus the PL lifetimes are prolonged (PdO/Pd–WO_3-4 and PdO/Pd–WO_3-5 samples), indicating that the extra PdO hinders the Schottky contact between Pd and WO_3 and is detrimental to charge separation.

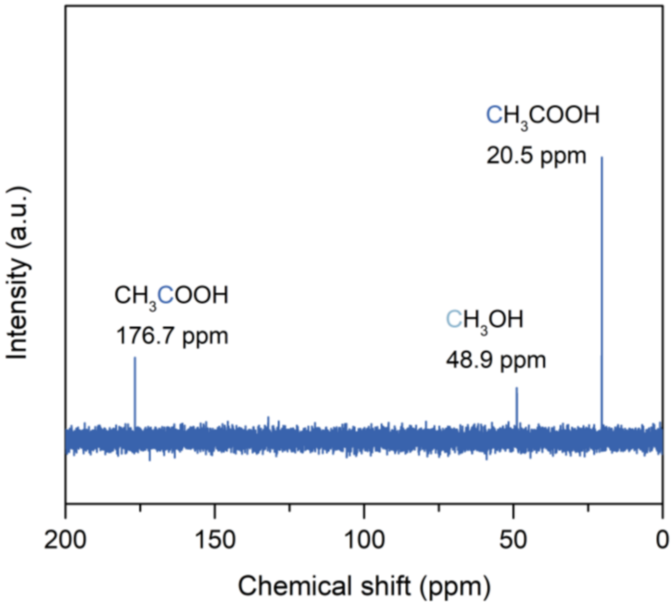

Supplementary Fig. 11 ^{13}C NMR spectrum of the produced CH_3COOH and CH_3OH.

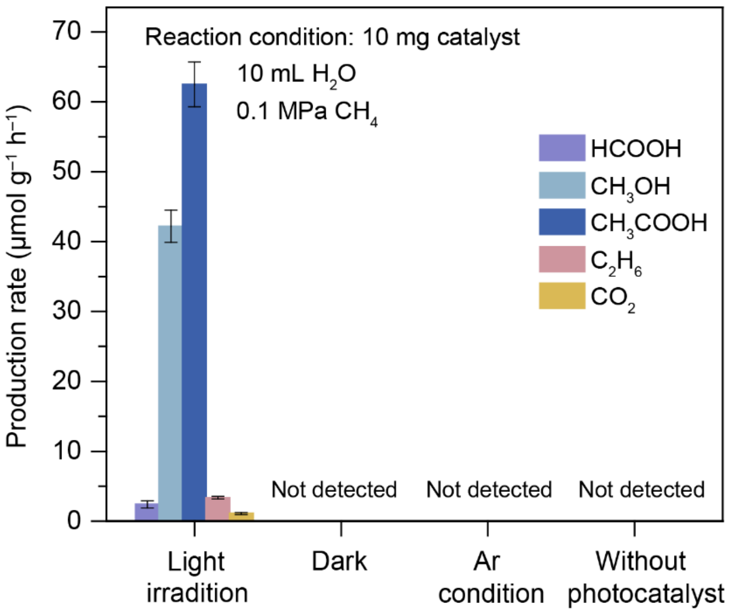

Supplementary Fig. 12 The comparison of photochemical CH_4 conversion performance over PdO/Pd–WO_3-2 in control experiments.

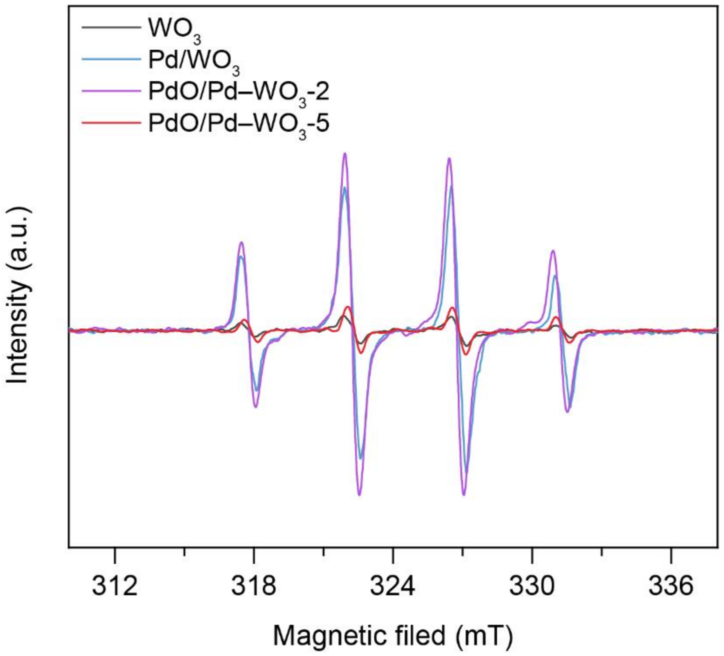

Supplementary Fig. 13 EPR spectra for ·OH radical production over the nanocomposites in water under light irradiation, using DMPO as a trapping agent.

EPR measurement is performed to compare the concentrations of produced ·OH radicals over the typical WO_3, Pd/WO_3, PdO/Pd–WO_3-2 and PdO/Pd–WO_3-5 nanocomposites under light irradiation. The ESR spectra display a 1:2:2:1 quadruplet signal, suggesting that the appropriate Pd/PdO ratio can significantly improve the generation of ·OH radicals[1]. However, with the further increase of PdO content, PdO will directly contact WO_3 to form PdO–WO_3 interface. As a result, the light-driven ·OH production is substantially suppressed due to the sluggish photo-induced charge separation at the PdO–WO_3 interface.

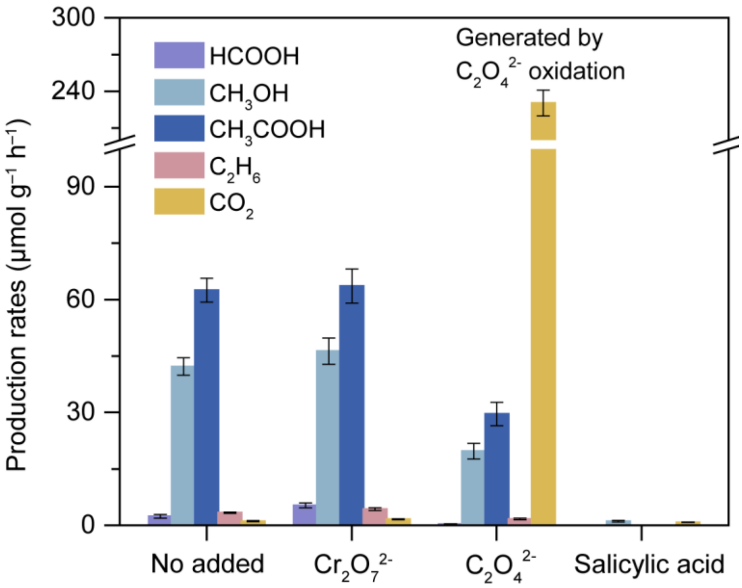

Supplementary Fig. 14 Production rates of photochemical CH_4 conversion over PdO/Pd–WO_3-2 with 1 mM $Cr_2O_7^{2-}$, $C_2O_4^{2}$- and salicylic acid added as photoinduced electron, hole and ·OH scavengers, respectively.

As shown in Supplementary Fig. 14, introducing $Cr_2O_7^{2-}$ as electron scavenger does not affect the production rates. However, the addition of $C_2O_4^{2-}$ as hole scavenger or salicylic acid as ·OH scavenger reduces the production rates significantly, indicating that the CH_4 photooxidation process is triggered by ·OH radicals.

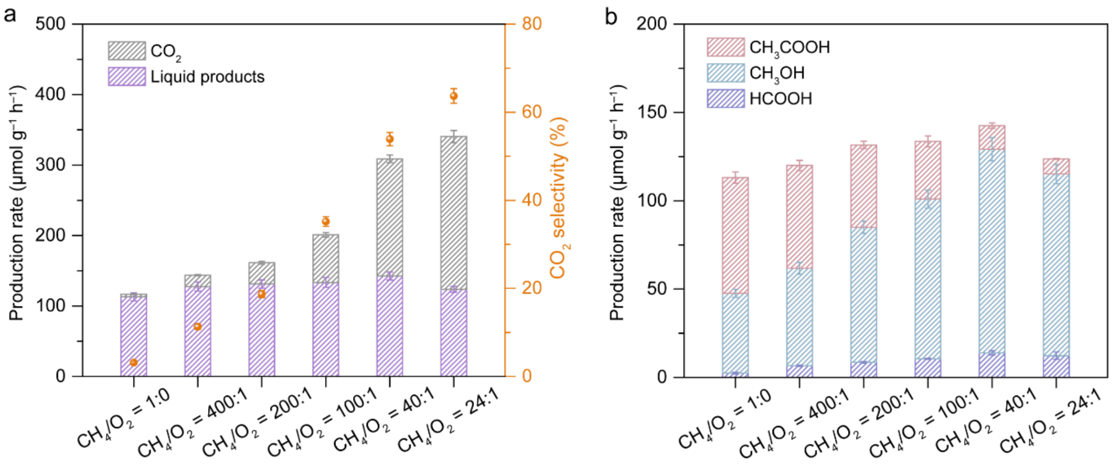

Supplementary Fig. 15 (a) The comparison of liquid products and CO_2 yield over PdO/Pd–WO_3-2 sample with different CH_4/O_2 ratio for CH_4 conversion under light irradiation. (b) Production rates of the liquid products over PdO/Pd–WO_3-2 sample with different CH_4/O_2 ratio for photochemical CH_4 conversion.

Introducing O_2 into reactants will not increase the total amount of produced liquid products but lead to CO_2 production. Among the liquid products, the CH_3OH production and selectivity are promoted. According to the previous work, O_2 can be activated on metal site to form superoxide radicals, which can oxidize CH_4 into CO_2 and CH_3OH[2].

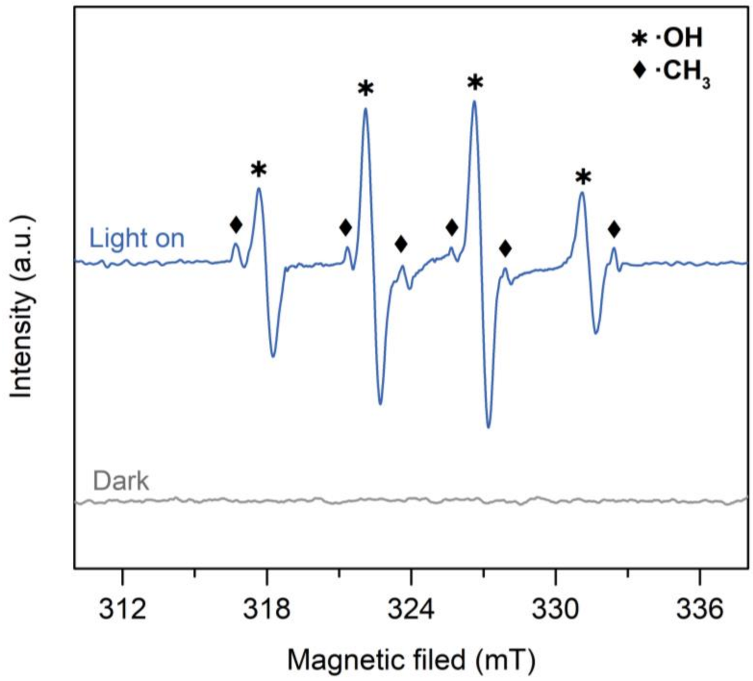

Supplementary Fig. 16 EPR spectra for the radical production over PdO/Pd–WO_3-2 sample in water with CH_4 as filling gas under light irradiation, using DMPO as a trapping agent.

As revealed by the EPR spectra, the signals of 1:2:2:1 quadruplet pattern are assigned to ·OH, and the sextuplet pattern near the signals of ·OH are ascribed to ·CH_3[3]. The results demonstrate the dissociation of CH_4 on PdO/Pd–WO_3-2 with light illumination.

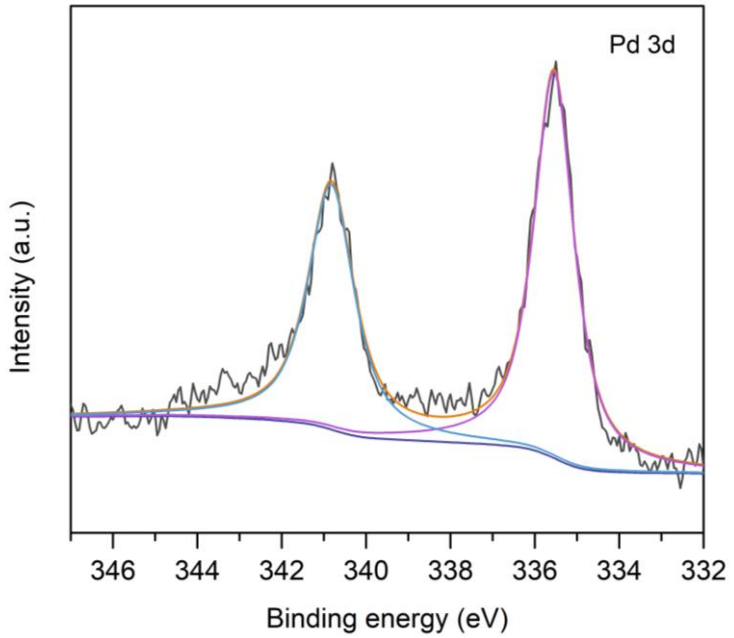

Supplementary Fig. 17 Pd 3*d* XPS spectrum of PdO/Pd–WO_3-2 sample after 10 h photochemical CH_4 conversion.

We perform the CH_4 conversion measurement for 10 h to investigate the variation of materials component. The result of Pd 3*d* XPS spectrum indicates that a great amount of PdO is consumed after the measurement, which may lead to the inactivation of nanocomposite.

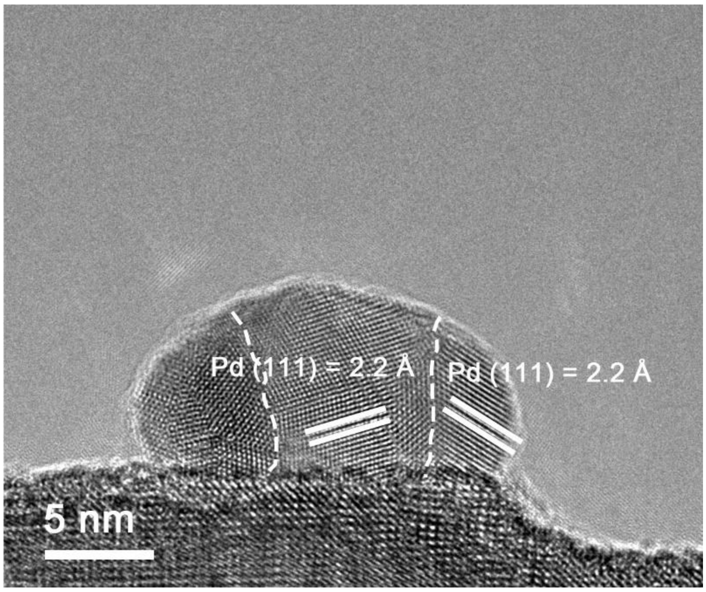

Supplementary Fig. 18 HRTEM image of PdO/Pd–WO_3-2 nanocomposite after long-time CH_4 conversion test.

The abundant grain boundary can be ascribed to the disintegration of Pd/PdO interface, suggesting that the PdO species is converted to Pd during the photochemical process.

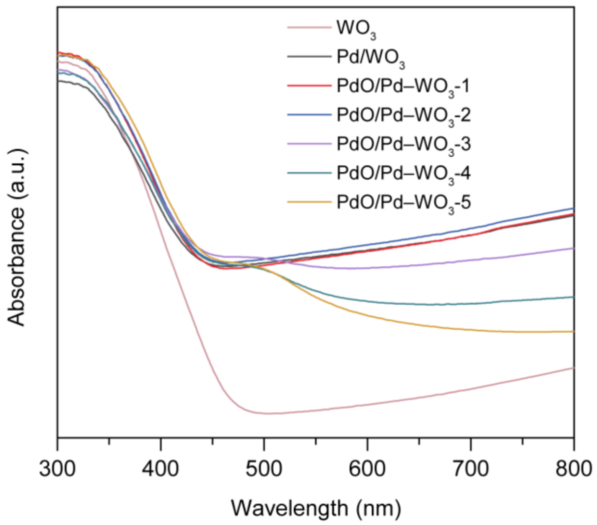

Supplementary Fig. 19 UV-vis diffuse reflectance spectra of the as-prepared samples.

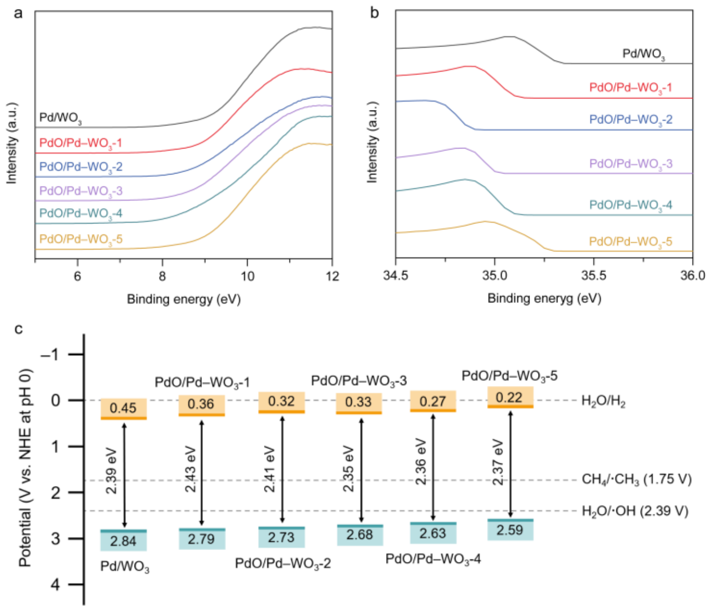

Supplementary Fig. 20 (a) Valence band spectra and (b) secondary electron cutoff for the as-prepared samples. The excitation photon energy is 40 eV. (c) The electronic band structures of the prepared samples.

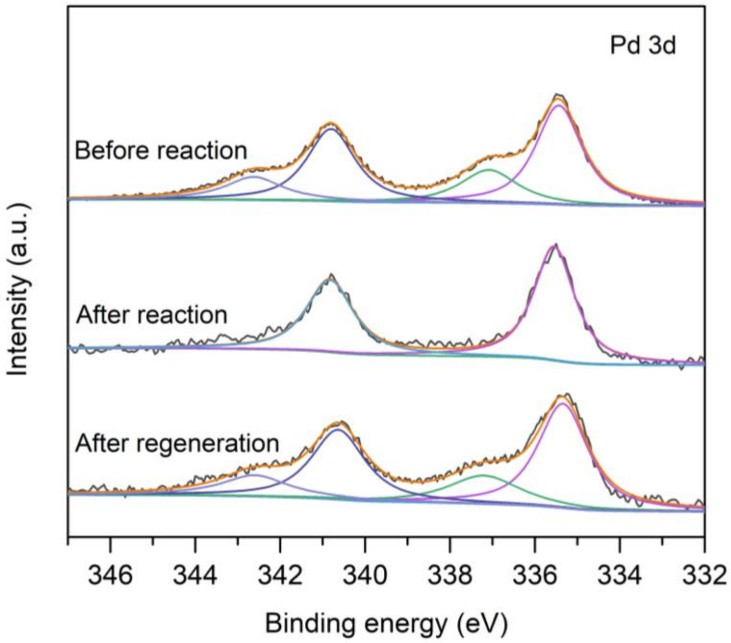

Supplementary Fig. 21 Pd 3*d* XPS spectra of PdO/Pd–WO_3-2 nanocomposite after the regeneration process in air.

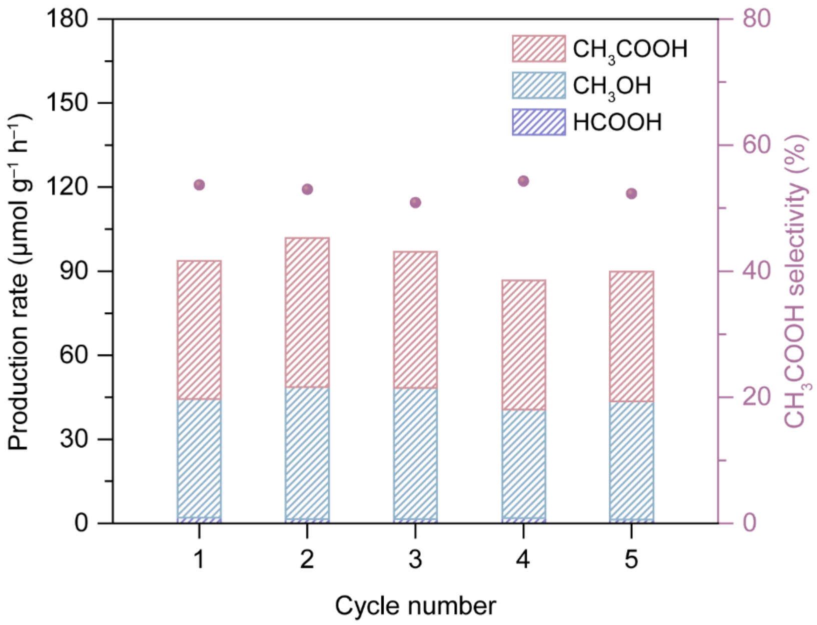

Supplementary Fig. 22 Production rates and selectivity for oxygenates in the cyclic tests by PdO/Pd–WO_3-2. Each cycle lasts 5 h, between which the nanocomposite is treated in air.

To perform the cyclic tests, 50 mg of nanocomposite is used in the process so that the collection of sample for regeneration treatments will be more convenient. Although the CH_4 conversion activity is related to the sample usage, our results show that the performance is approximate with the tests using 10 mg of sample in this work.

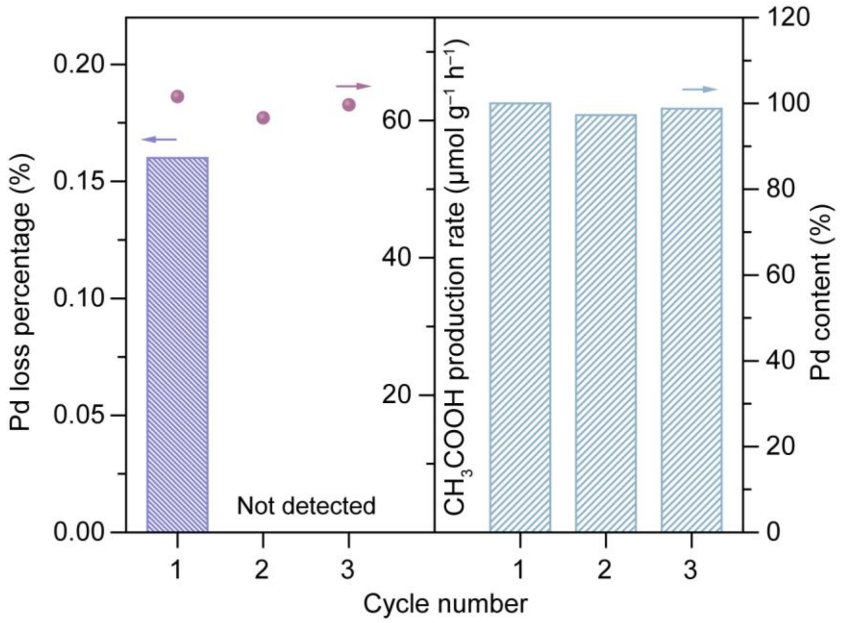

Supplementary Fig. 23 The Pd loss percentage detected by ICP-MS and the CH_3COOH production rates during cyclic tests (left side). The Pd content in material after cyclic tests is compared with the initial sample, which is detected by ICP-OES (right side).

As shown in Supplementary Fig. 23, Pd^{2+} can only be detected in the first round of reaction solution, corresponding to 0.16% Pd loss in the first cycle. During the measured three cyclic tests, the photochemical performance is well maintained, suggesting that the Pd atoms are stable in the PdO/Pd–WO_3 heterointerface. The total Pd content is measured by ICP-OES, showing that the Pd content after regeneration process is similar to the initial sample. It should be noted that ICP-MS is substantially more sensitive than ICP-OES.

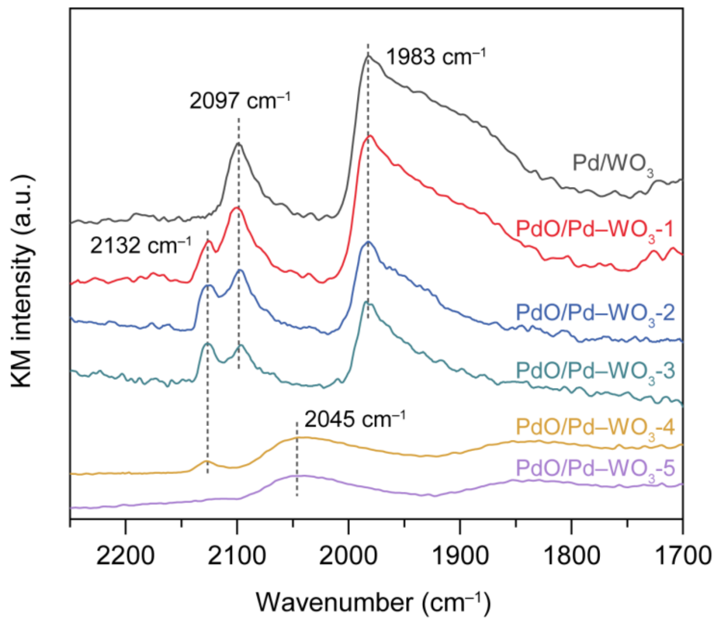

Supplementary Fig. 24 CO adsorption DRIFTS spectra of the as-prepared samples.

As shown in Supplementary Fig. 24, the peaks at 2097 and 1983 cm^{-1} are ascribed to the linear- and bridge-type CO adsorption on Pd site, respectively. The new peaks arising at 2132 cm^{-1} over PdO/Pd–WO_3-1 to PdO/Pd–WO_3-3 sample are attributed to the linear CO adsorption on partially oxidized Pd[4], demonstrating the well-formed Pd/PdO heterointerface in the samples. However, according to the report by Zorrn et al., the CO adsorption on fully oxidized Pd is weak[5]. This leads to the gradual disappearance of the peak at 2132 cm^{-1} over PdO/Pd–WO_3-4 and PdO/Pd–WO_3-5 samples. Of note, the broad peaks at 2045 cm^{-1} are observed, which can be assigned to bridge-type CO adsorption over PdO edges with WO_3 supports, corresponding to the detection result of carbonyl intermediate by in situ DRIFTS.

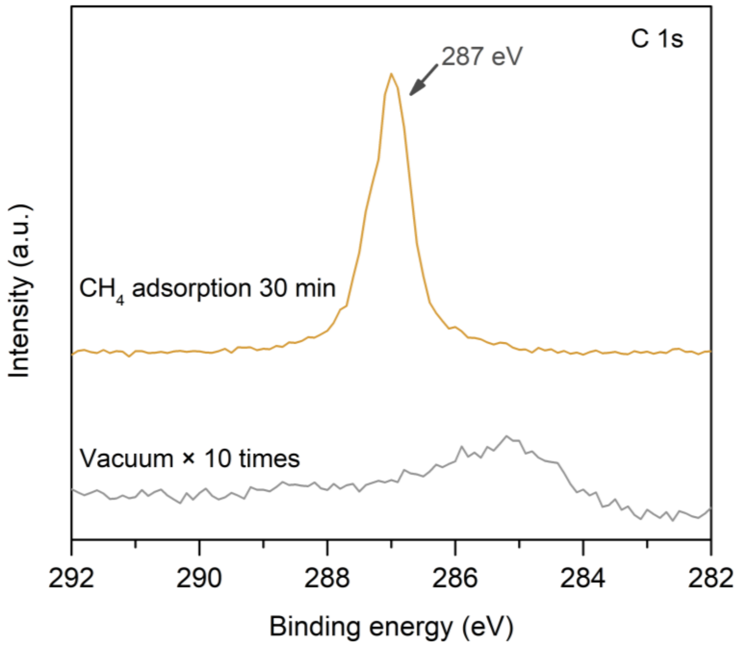

Supplementary Fig. 25 High-resolution C 1*s* XPS spectra of PdO/Pd–WO_3-2 sample under vacuum condition and after CH_4 adsorption for 30 min.

The surface-adsorbed carbon on PdO/Pd–WO_3-2 sample has been removed by the pretreatment before NAP-XPS measurements so that the intensity of the surface carbon impurity is too weak and can be ignored.

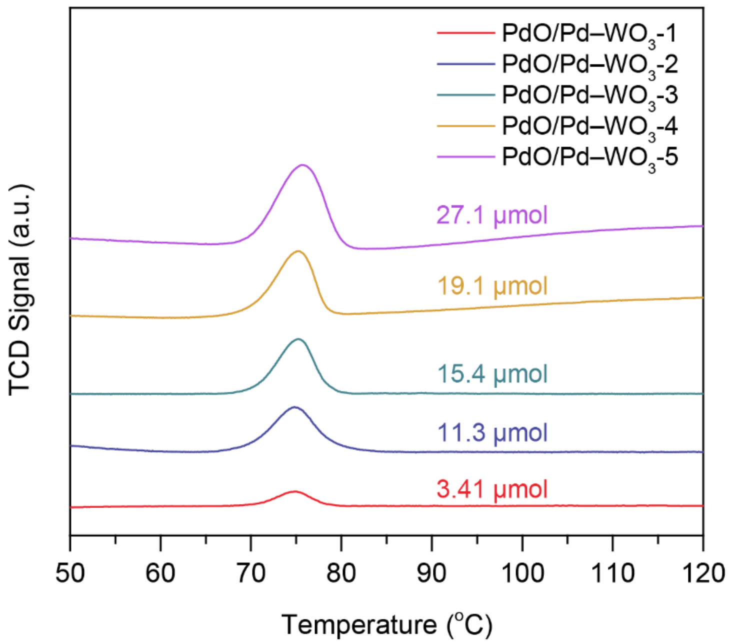

Supplementary Fig. 26 H_2–TPR analysis of PdO/Pd–WO_3-1 to PdO/Pd–WO_3-5 samples.

We perform H_2–TPR to quantify the PdO content in the as-prepared samples (50 mg). The peaks of H_2 consumption at 75 ^{o}C represent the complete reduction of PdO species on WO_3. As such, the content of PdO can be determined through calculating the corresponding H_2 consumption by quantitative relation (PdO:H_2 = 1:1).

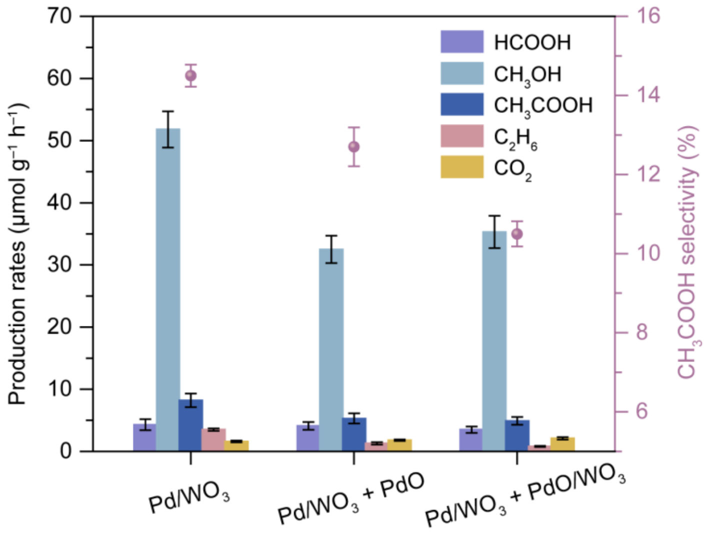

Supplementary Fig. 27 The comparison of photochemical CH_4 conversion over Pd/WO_3, the mixture of Pd/WO_3 with PdO and the mixture of Pd/WO_3 with PdO/WO_3. The content of mixed PdO is controlled to approach that in PdO/Pd–WO_3-2 sample.

The photochemical performance cannot be improved through simple physical mixing of Pd/WO_3 composite with PdO, further corroborating the importance of Pd/PdO interface to CH_3COOH synthesis.

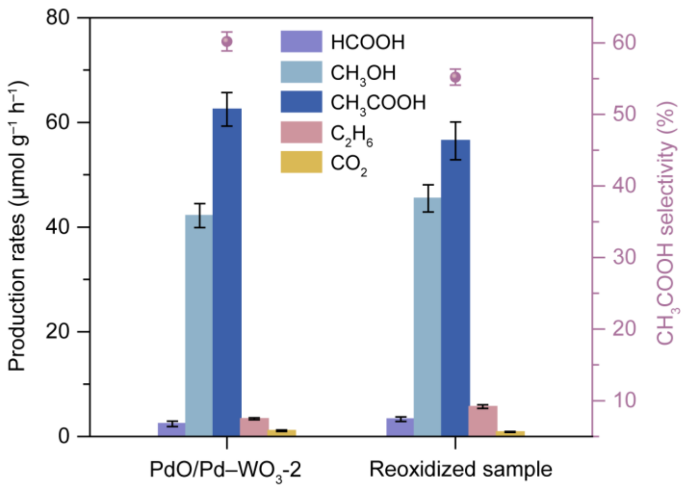

Supplementary Fig. 28 The comparison of photochemical CH_4 conversion performance over the reoxidized sample and PdO/Pd–WO_3-2.

The reoxidation of the reduced PdO/Pd–WO_3-2 sample can roughly achieve the performance of fresh PdO/Pd–WO_3-2, implying that the construction of appropriate Pd/PdO interface is favorable for CH_3COOH production

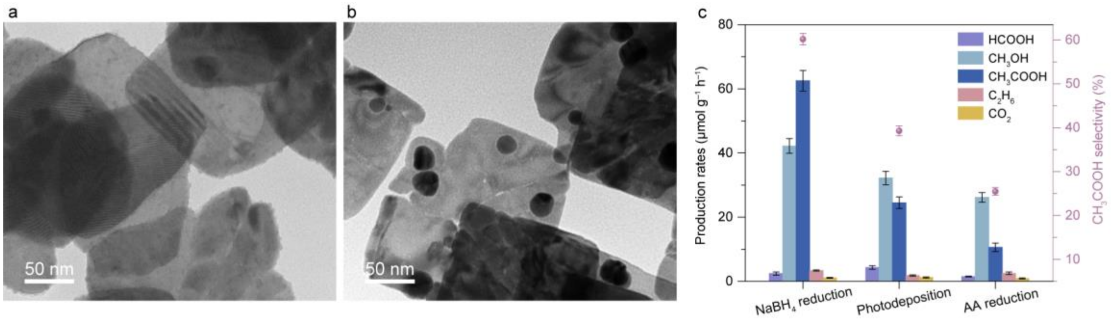

Supplementary Fig. 29 TEM images of PdO/Pd–WO_3-2 samples synthesized by (a) photo-deposition and (b) ascorbic acid (AA) reduction with different particle sizes. (c) The photochemical CH_4 conversion performance of the as-prepared PdO/Pd–WO_3-2 samples.

It should be noted that the photochemical performance is related to the size of nanoparticles by tailoring their oxidation degree in the calcination process. We further regulate the Pd nanoparticle size by changing the synthesis process. With the same annealing treatment on PdO/Pd–WO_3-2, the lower photochemical properties are observed in both the nanocomposites with particles size of 2 nm and 27 nm, indicating that the main structural character is the optimized Pd/PdO interface.

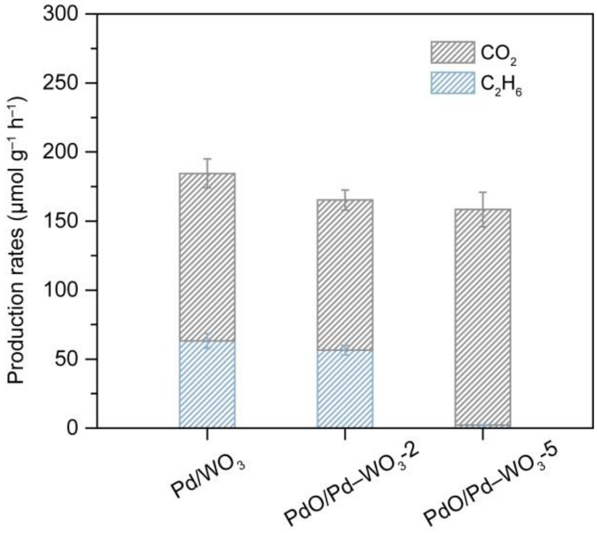

Supplementary Fig. 30 The light-driven nonoxidative coupling of methane (NOCM) performance over the typical Pd/WO_3, PdO/Pd–WO_3-2 and PdO/Pd–WO_3-5 samples in 0.1 MPa CH_4.

Generally, Pd^0 can generate and stabilize *CH_3 for *CH_3 coupling[6], while CH_4 undergoes carbonylation and combustion ($CH_4 \rightarrow CH_3O \rightarrow \ldots \rightarrow CO_2$) over Pd^{2+} (PdO) site through Mar–van Krevelen mechanism[7]. To further validate their role in PdO/Pd–WO_3-2, we perform the light-driven nonoxidative coupling of methane over the three typical samples. As shown in Supplementary Fig. 30, the samples exhibit the order of Pd/WO_3 > PdO/Pd–WO_3-2 > PdO/Pd–WO_3-5 for C_2H_6 production. In particular, substantially more CO_2 is detected over the PdO/Pd–WO_3-5 sample while negligible C_2H_6 is observed, implying that the Pd^0 on PdO/Pd–WO_3 is the predominant active species for *CH_3 generation as compared with Pd^{2+}.

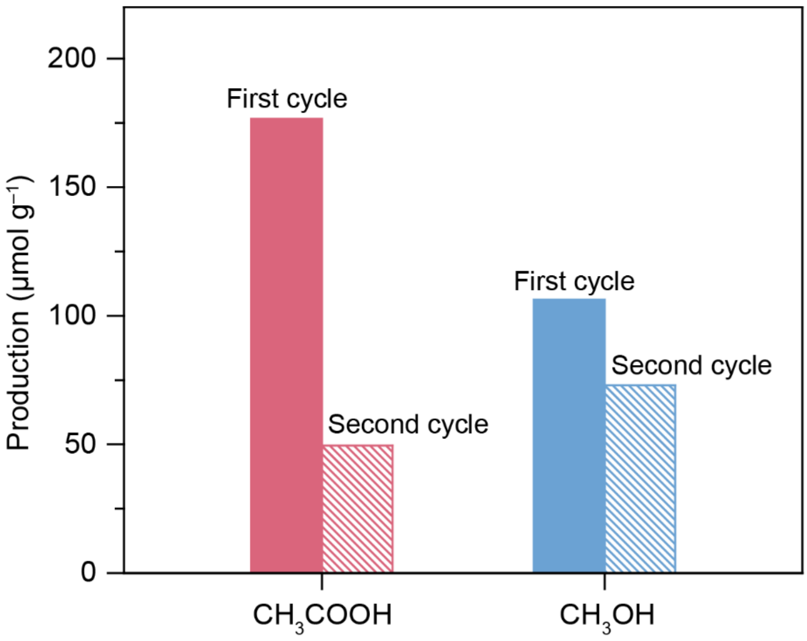

Supplementary Fig. 31 The comparison of CH_3COOH and CH_3OH production in the cyclic tests over PdO/Pd–WO_3-2 without regeneration treatment. Each cycle last 3 h.

Of note, the CH_3OH production is derived from ·OH radical, which is associated with the separation efficiency of photogenerated electrons and holes. Without the regeneration process, the CH_3COOH production exhibits dramatically declined in the second cycle while the CH_3OH production maintains 80%. This indicates that the activity especially for CH_3COOH production is more influenced by the consumption of lattice oxygen in PdO.

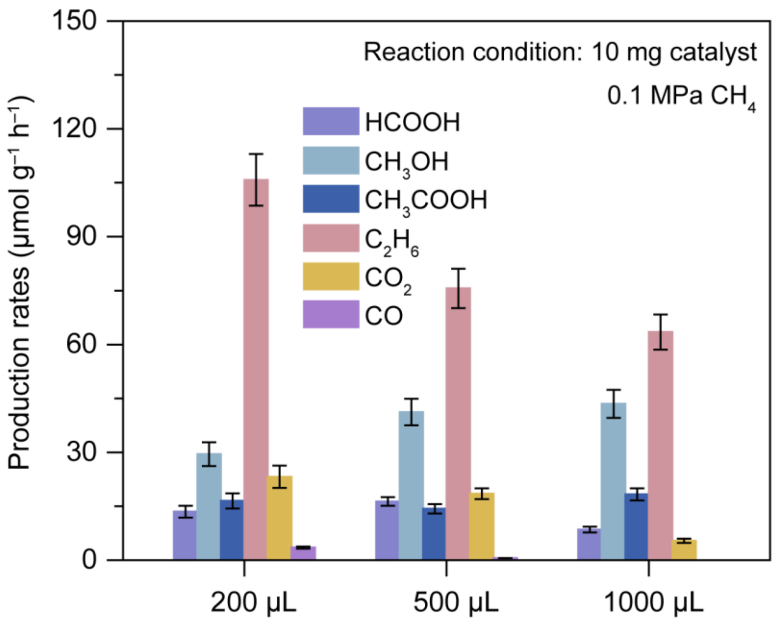

Supplementary Fig. 32 Photochemical CH_4 conversion performance over PdO/Pd–WO_3-2 in gas–solid phase with different H_2O usage.

In the control experiments, the reactants are CH_4 and water vapor, in which the generated $\cdot CH_3$ is more able to undergo coupling in gas phase. Improving H_2O content promotes oxygenate production, but CH_4 coupling can still be obviously observed. Therefore, the flux of H_2O in reaction system is important for enhancing $\cdot CH_3$ and *CO utilization.

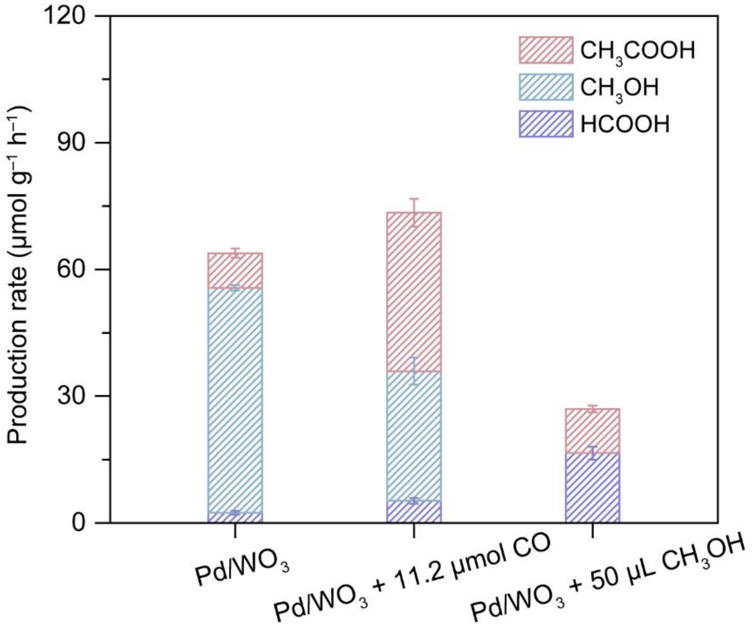

Supplementary Fig. 33 The comparison of liquid products over Pd/WO_3 sample with pure CH_4, CH_4/CO and CH_4/CH_3OH as reactants for photochemical CH_4 conversion.

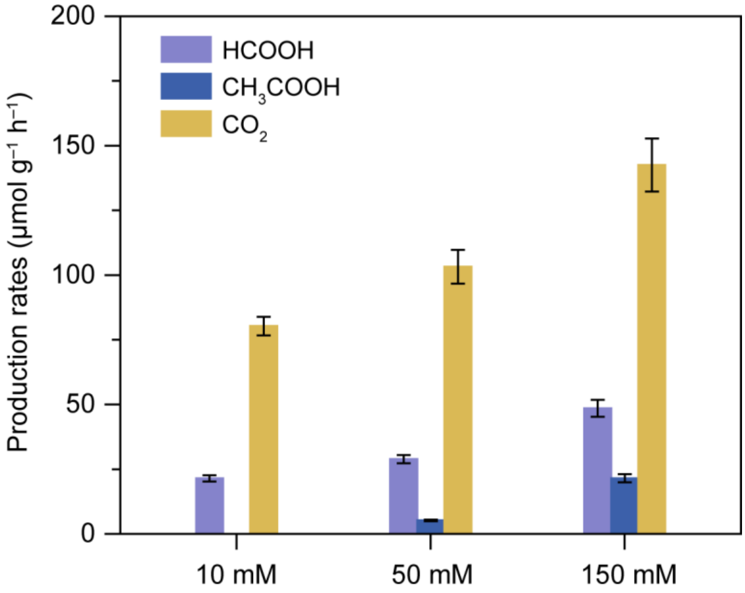

Supplementary Fig. 34 The comparison of photochemical methanol oxidation performance over PdO/Pd–WO_3-2 with different methanol concentrations under Ar condition.

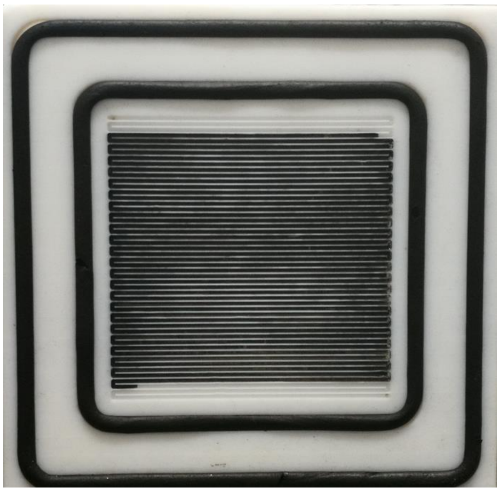

Supplementary Fig. 35 The photograph of homemade photochemical reactor with arc-shaped flow channel. The ash black component is sample loading.

Supplementary Table 1. The corresponding contents of Pd in the prepared samples. The content of Pd in the samples is determined by inductively coupled plasma-optical emission spectrometry (ICP-OES).

Sample	Content of Pd
Pd/WO_3	6.25 wt.%
$PdO/Pd–WO_3$-1	6.82 wt.%
$PdO/Pd–WO_3$-2	6.57 wt.%
$PdO/Pd–WO_3$-3	6.32 wt.%
$PdO/Pd–WO_3$-4	6.71 wt.%
$PdO/Pd–WO_3$-5	6.39 wt.%

Supplementary Table 2. Representative works on light-driven CH_4 conversion under mild condition.

Samples	CH_4 conversion rate	Product	Production rate	Selectivity	Reference
PdO/Pd–WO_3-2 in batch reactor	181.5 μmol g^{-1} h^{-1}	CH_3COOH	62.5 μmol g^{-1} h^{-1}	60.2%	This work
PdO/Pd–WO_3-2 in flow reactor	190.3 μmol g^{-1} h^{-1}	CH_3COOH	90.7 μmol g^{-1} h^{-1}	91.6%	This work
Cu-0.5/PCN	236.5 μmol g^{-1} h^{-1}	CH_3CH_2OH	106 μmol g^{-1} h^{-1}	81.2%	[8]
ZnO/Fe_2O_3	118.84 μmol g^{-1} h^{-1}	CH_3OH	118.84 μmol g^{-1} h^{-1}	99.6 %	[9]
La-WO_3	76 μmol g^{-1} h^{-1}	CH_3OH	36.7 μmol g^{-1} h^{-1}	46%	[10]
Bi_2WO_6	51 μmol g^{-1} h^{-1}	CH_3OH	15.0 μmol g^{-1} h^{-1}	29.3%	[11]
$BiVO_4$	132 μmol g^{-1} h^{-1}	CH_3OH	111.9 μmol g^{-1} h^{-1}	85%	[12]
Mesoporous WO_3	147 μmol g^{-1} h^{-1}	CH_3OH	55.5 μmol g^{-1} h^{-1}	37.4%	[13]

Supplementary References

1. Song, H. et al. Direct and selective photocatalytic oxidation of CH_4 to oxygenates with O_2 on cocatalysts/ZnO at room temperature in water. *J. Am. Chem. Soc.* **141,** 20507-20515 (2019).
2. Song, H. et al. Selective photo-oxidation of methane to methanol with oxygen over dual-cocatalyst-modified titanium dioxide. *ACS Catal.* **10,** 14318-14326 (2020).
3. Shen, Q. et al. Single chromium atoms supported on titanium dioxide nanoparticles for synergic catalytic methane conversion under mild conditions. *Angew. Chem. Int. Ed.* **59,** 1216-1219 (2020).
4. Jang, E. J., Lee, J., Oh, D. G. & Kwak, J. H. CH_4 oxidation activity in Pd and Pt–Pd bimetallic catalysts: correlation with surface PdO_x quantified from the DRIFTS study. *ACS Catal.* **11,** 5894-5905 (2021).
5. Zorn, K. et al. CO oxidation on technological Pd−Al_2O_3 catalysts: oxidation state and activity. *J. Phys. Chem. C* **115,** 1103-1111 (2011).
6. Singh, S. P. et al. A Pd-Bi dual-cocatalyst-loaded gallium oxide photocatalyst for selective and stable nonoxidative coupling of methane. *ACS Catal.* **11,** 13768-13781 (2021).
7. Feng, X. et al. Highly active PdO/Mn_3O_4/CeO_2 nanocomposites supported on one dimensional halloysite nanotubes for photoassisted thermal catalytic methane combustion. *Angew. Chem. Int. Ed.* **60,** 18552-18556 (2021).
8. Zhou, Y., Zhang, L. & Wang, W. Direct functionalization of methane into ethanol over copper modified polymeric carbon nitride via photocatalysis. *Nat. Commun.* **10,** 506 (2019).
9. Zheng, K. et al. Room-temperature photooxidation of CH_4 to CH_3OH with nearly 100% selectivity over hetero-ZnO/Fe_2O_3 porous nanosheets. *J. Am. Chem. Soc.* **144,** 12357-12366 (2022).
10. Villa, K., Murcia-López, S., Morante, J. R. & Andreu, T. An insight on the role of La in mesoporous WO_3 for the photocatalytic conversion of methane into methanol. *Appl. Catal. B* **187,** 30-36 (2016).
11. Murcia-López, S., Villa, K., Andreu, T. & Morante, J. R. Partial oxidation of methane to methanol using bismuth-based photocatalysts. *ACS Catal.* **4,** 3013-3019 (2014).
12. Zhu, W. et al. Facet-dependent enhancement in the activity of bismuth vanadate microcrystals for the photocatalytic conversion of methane to methanol. *ACS Appl. Nano Mater.* **1,** 6683-6691 (2018).
13. Villa, K., Murcia-López, S., Andreu, T. & Morante, J. R. Mesoporous WO_3 photocatalyst for the partial oxidation of methane to methanol using electron scavengers. *Appl. Catal. B* **163,** 150-155 (2015).
